# Association of Timing of Epinephrine Administration With Outcomes in Adults With Out-of-Hospital Cardiac Arrest

**DOI:** 10.1001/jamanetworkopen.2021.20176

**Published:** 2021-08-10

**Authors:** Masashi Okubo, Sho Komukai, Clifton W. Callaway, Junichi Izawa

**Affiliations:** 1 Department of Emergency Medicine, University of Pittsburgh School of Medicine, Pittsburgh, Pennsylvania; 2 Division of Biomedical Statistics, Department of Integrated Medicine, Osaka University Graduate School of Medicine, Suita, Japan; 3 Department of Internal Medicine, Okinawa Prefectural Yaeyama Hospital, Okinawa, Japan

## Abstract

**Importance:**

Administration of epinephrine has been found to be associated with an increased chance of survival after out-of-hospital cardiac arrest (OHCA), but the optimal timing of administration has not been fully investigated.

**Objective:**

To ascertain whether there is an association between timing of epinephrine administration and patient outcomes after OHCA.

**Design, Setting, and Participants:**

This cohort study included adults 18 years or older with OHCA treated by emergency medical services (EMS) personnel from April 1, 2011, to June 30, 2015. Initial cardiac rhythm was stratified as either initially shockable (ventricular defibrillation or pulseless ventricular tachycardia) or nonshockable (pulseless electrical activity or asystole). Eligible individuals were identified from among publicly available, deidentified patient-level data from the Resuscitation Outcomes Consortium Cardiac Epidemiologic Registry, a prospective registry of adults with EMS-treated, nontraumatic OHCA with 10 sites in North America. Data analysis was conducted from May 2019 to April 2021.

**Exposures:**

Interval between advanced life support (ALS)–trained EMS personnel arrival at the scene and the first prehospital intravenous or intraosseous administration of epinephrine.

**Main Outcomes and Measures:**

The primary outcome was survival to hospital discharge. In each cohort of initial cardiac rhythms, patients who received epinephrine at any period (minutes) after EMS arrival at the scene were matched with patients who were at risk of receiving epinephrine within the same period using time-dependent propensity scores calculated from patient demographic characteristics, arrest characteristics, and EMS interventions.

**Results:**

Of 41 079 eligible individuals (median [interquartile range] age, 67 [55-79] years), 26 579 (64.7%) were men. A total of 10 088 individuals (24.6%) initially had shockable cardiac rhythms, and 30 991 (75.4%) had nonshockable rhythms. Those who received epinephrine included 8223 patients (81.5%) with shockable cardiac rhythms and 27 901 (90.0%) with nonshockable rhythms. In the shockable cardiac rhythm cohort, the risk ratio (RR) for receipt of epinephrine with survival to hospital discharge was highest between 0 and 5 minutes after EMS arrival (1.12; 95% CI, 0.99-1.26) across the categorized timing of the administration of epinephrine by 5-minute intervals after EMS arrival; however, that finding was not statistically significant. Treating the timing of epinephrine administration as a continuous variable, the RR for survival to hospital discharge decreased 5.5% (95% CI, 3.4%-7.5%; *P* < .001 for the interaction between epinephrine administration and time to matching) per minute after EMS arrival. In the nonshockable cardiac rhythm cohort, the RR for the association of receipt of epinephrine with survival to hospital discharge was the highest between 0 and 5 minutes (1.28; 95% CI, 0.95-1.72), although not statistically significant, and decreased 4.4% (95% CI, 0.8%-7.9%; *P* for interaction = .02) per minute after EMS arrival.

**Conclusions and Relevance:**

Among adults with OHCA, survival to hospital discharge differed across the timing of epinephrine administration and decreased with delayed administration for both shockable and nonshockable rhythms.

## Key Points

**Question:**

Is timing of epinephrine administration associated with outcomes in adults with out-of-hospital cardiac arrest?

**Findings:**

In this cohort study with time-dependent propensity score and risk-set matching analysis of 41 079 adult patients from a large out-of-hospital cardiac arrest registry in the United States and Canada, survival to hospital discharge and favorable functional status at hospital discharge were statistically significant and differed according to the timing of epinephrine administration, and the risk ratios for survival and favorable functional status decreased with delayed administration of epinephrine.

**Meaning:**

Findings of this study suggest that early epinephrine administration is associated with better survival outcomes in adult patients with shockable and nonshockable out-of-hospital cardiac arrest.

## Teaser

This cohort study evaluates survival and functional status outcomes associated with timing of epinephrine administration in adults with out-of-hospital cardiac arrest with or without initial shockable cardiac rhythms.

## Introduction

Out-of-hospital cardiac arrest (OHCA) is a major public health problem with high mortality, affecting more than 350 000 individuals annually in the United States.^1^ Intravenous and intraosseous administration of epinephrine has been widely used for OHCA in the prehospital setting.^2^ In a recent randomized clinical trial, the use of epinephrine in adults with OHCA increased survival.^3^ However, evidence about the optimal timing of epinephrine administration is insufficient.^4^ The 2020 International Consensus on Cardiopulmonary Resuscitation and Emergency Cardiovascular Care Science With Treatment Recommendations^5^ and 2020 American Heart Association Guidelines for Cardiopulmonary Resuscitation and Emergency Cardiovascular Care^6^ recommend administration of epinephrine as soon as feasible for individuals with nonshockable cardiac rhythms (strong recommendation with a low certainty of evidence) and suggest administration of epinephrine after initial defibrillation attempts are unsuccessful for shockable cardiac rhythms (weak recommendation with low certainty of evidence). The low certainty of evidence for these recommendations suggests that the optimal timing of epinephrine administration is an existing knowledge gap.^4^

A 2019 systematic review indicated that previous studies evaluating the timing of epinephrine had inconsistent findings and a critical risk of bias.^7^ Notably, none of the included studies addressed an essential factor: resuscitation time bias.^8^ When timing of an intra-arrest intervention (eg, epinephrine) is assessed, it is crucial to account for this bias.^8^ Patients cannot achieve return of spontaneous circulation (ROSC) before the intra-arrest intervention.^8^ Therefore, the late intervention group tends to have longer resuscitation duration and is biased toward harm compared with the early intervention group because longer resuscitation duration is associated with worse outcomes.^9,10^

One approach to address resuscitation time bias and time-varying confounders is a time-dependent propensity score and risk-set matching analysis,^11,12,13,14,15^ which, to our knowledge, has not been used to assess the timing of epinephrine administration for OHCA. The aim of the present study was to use this approach to ascertain whether the timing of epinephrine administration was associated with survival and functional outcomes in patients with OHCA.

## Methods

### Study Design and Setting

The Resuscitation Outcomes Consortium (ROC) was a clinical research network that conducted trials in OHCA at 10 regional coordinating sites across North America.^16,17^ In this cohort study, we performed a secondary analysis of patients included in the ROC Cardiac Epidemiologic Registry, a database of prospectively identified, consecutive patients with EMS-treated OHCA (April 2011 through June 2015).^16,17^ We obtained the publicly available, deidentified patient-level data from the National Heart, Lung, and Blood Institute.^18^ The institutional review boards at the University of Pittsburgh and Osaka University approved this study and waived the requirement for informed consent because publicly available deidentified data were used. We followed the Strengthening the Reporting of Observational Studies in Epidemiology (STROBE) reporting guideline.

### Study Participants

Included in the study were adults 18 years or older with EMS-treated, non-traumatic OHCA defined as initiation of resuscitation attempts with shock delivery by an external defibrillator (by layperson or EMS personnel) or chest compression by EMS personnel.^17^ We excluded patients with EMS-witnessed OHCA; those without advanced life support (ALS) involvement; those in whom resuscitations were terminated in the prehospital setting because of confirmation of preexisting written do-not-resuscitate orders; those with missing data on age, initial cardiac rhythm, epinephrine administration status, and primary outcome; those who received vasopressin or endotracheal epinephrine administration; and those with missing or negative values in resuscitation time variables. Resuscitation time variables included intervals between the 9-1-1 call and the first EMS vehicle arrival, between ALS arrival and shock delivery by ALS-trained EMS personnel (if an ALS-trained EMS personnel delivered the shock), between ALS arrival and the first epinephrine administration (if a patient received epinephrine), between ALS arrival and advanced airway management (if a patient received advanced airway management), between ALS arrival and departure from the scene (if a patient was transported), between ALS arrival and prehospital ROSC (if a patient had ROSC), between ALS arrival and prehospital termination of resuscitation (TOR) (if a patient had TOR), and between ALS arrival and hospital arrival (if a patient was transported).

### Exposure and Outcomes

The main exposure was the interval between ALS-trained EMS personnel arrival at the scene and the first prehospital intravenous or intraosseous administration of epinephrine. The interval was defined in whole minutes; therefore, epinephrine administration at 0 minute indicates that the patient received epinephrine within the same whole minute that EMS arrived.

The primary outcome was survival to hospital discharge. Secondary outcomes were favorable functional status at hospital discharge, which was defined as a modified Rankin Scale score of 3 or lower, and prehospital ROSC.

### Statistical Analysis

We stratified patients into 2 cohorts based on their initial cardiac rhythms—shockable (ventricular defibrillation or pulseless ventricular tachycardia) or nonshockable (pulseless electrical activity or asystole) rhythms—because current resuscitation guidelines recommend 2 treatment algorithms according to the initial cardiac rhythms.^6,19^ In this deidentified data set, age 89 years or older was not specified; therefore, we coded any age 89 years or older as 89 years. We performed multiple imputations to address missing data for functional outcomes, assuming missing at random^20^; 20 imputed data sets were created through this process, which was conducted after risk-set matching. The regression coefficients for the separately analyzed imputed data sets were averaged and the variances were estimated using mathematical rules as described by Newgard and Haukoos.^20^ We rounded decimal places to use whole numbers when imputing the number of patients with favorable functional status.

To assess for an association between the timing of epinephrine administration and outcomes, we performed a time-dependent propensity score and risk-set matching analysis in each cohort of initial cardiac rhythms.^11,12,13,14,15,21,22,23^ We calculated the propensity score as the time-varying probability of receiving epinephrine using a Fine-Gray regression model.^13,14,15,24^ In the survival analysis model, time to receipt of the first epinephrine administration was the dependent variable, and EMS arrival was time 0 because patients were at risk of receiving epinephrine only after this period. We included the covariates (eg, location, witnessed collapse, and cardiopulmonary resuscitation performed by a bystander) shown in Table 1. Additional methodological details are provided in the eMethods in the Supplement.

**Table 1.** Characteristics and Covariates of Adults With Out-of-Hospital Cardiac Arrest With and Without Epinephrine in Original Cohort^a^

Characteristic or Covariate	Shockable cardiac rhythms			Nonshockable cardiac rhythms		
	No epinephrine (n = 1865)	Epinephrine (n = 8223)	Standardized difference	No epinephrine (n = 3090)	Epinephrine (n = 27 901)	Standardized difference
Age, median (IQR), y	62 (53-72)	65 (55-76)	0.19	69 (56-82)	68 (55-80)	0.05
Sex						
Male	1342 (72.0)	6414 (78.0)	0.14	1712 (55.4)	17 111 (61.3)	0.12
Female	522 (28.0)	1805 (22.0)		1376 (44.5)	10 778 (38.6)	
Unknown	1 (0.1)	4 (0.0)		2 (0.1)	12 (0.0)	
Race						
White	484 (26.0)	2085 (25.4)	0.01	617 (20.0)	6842 (24.5)	0.11
Non-White^b^	1381 (74.0)	6138 (74.6)		2473 (80.0)	21 059 (75.5)	
Cause						
Cardiac	1824 (97.8)	8076 (98.2)	0.04	2692 (87.1)	25 814 (92.5)	0.18
Noncardiac	40 (2.1)	146 (1.8)		397 (12.8)	2087 (7.5)	
Unknown	1 (0.1)	1 (0.0)		1 (0.0)	0 (0.0)	
Initial rhythm						
PEA (shockable)	NA	NA		1306 (42.3)	8252 (29.6)	0.27
Asystole (nonshockable)	NA	NA		1784 (57.7)	19 649 (70.4)	
Location of cardiac arrest						
Street/highway	216 (11.6)	747 (9.1)	0.28	120 (3.9)	793 (2.8)	0.12
Public building	64 (3.4)	170 (2.1)		25 (0.8)	177 (0.6)	
Place of recreation	131 (7.0)	342 (4.2)		54 (1.7)	325 (1.2)	
Industrial place	49 (2.6)	179 (2.2)		3 (0.1)	133 (0.5)	
Home	977 (52.4)	5320 (64.7)		2283 (73.9)	21 109 (75.7)	
Farm/ranch	0 (0.0)	7 (0.1)		3 (0.1)	26 (0.1)	
Health care facility	41 (2.2)	133 (1.6)		75 (2.4)	566 (2.0)	
Residential institution	64 (3.4)	300 (3.6)		355 (11.5)	3436 (12.3)	
Other public property	294 (15.8)	956 (11.6)		157 (5.1)	1200 (4.3)	
Other nonpublic property	21 (1.1)	49 (0.6)		6 (0.2)	84 (0.3)	
Unknown	8 (0.4)	20 (0.2)		9 (0.3)	52 (0.2)	
Witnessed collapse						
Bystander	1474 (79.0)	5344 (65.0)	0.33	1151 (37.2)	9435 (33.8)	0.07
None	348 (18.7)	2693 (32.7)		1867 (60.4)	17 691 (63.4)	
Unknown	43 (2.3)	186 (2.3)		72 (2.3)	775 (2.8)	
Layperson CPR						
Yes	1281 (68.7)	4683 (57.0)	0.25	1338 (43.3)	13 181 (47.2)	0.08
No	549 (29.4)	3395 (41.3)		1691 (54.7)	14 120 (50.6)	
Unknown	35 (1.9)	145 (1.8)		61 (2.0)	600 (2.2)	
Shock delivery before arrival of ALS-trained EMS personnel						
Yes	715 (38.3)	2383 (29.0)	0.22	35 (1.1)	306 (1.1)	0.04
No	1150 (61.7)	5840 (71.0)		3055 (98.9)	27 595 (98.9)	
EMS response time (interval between 9-1-1 call and first EMS arrival), median (IQR), min	5.1 (4.0-6.6)	5.5 (4.3-7.0)	0.16	5.5 (4.3-7.0)	5.4 (4.2-7.0)	0.07
Shock delivery after ALS arrival						
Yes	1139 (61.1)	5676 (69.0)	0.17	161 (5.2)	4489 (16.1)	0.36
Interval between ALS arrival and shock delivery, median (IQR), min	3.2 (2.0-4.6)	4.0 (2.5-5.9)	0.27	10.3 (4.1-18.1)	13.5 (8.5-19.9)	0.28
Advanced airway management						
Yes	851 (45.6)	6680 (81.2)	0.80	967 (31.3)	21 899 (78.5)	1.07
Interval between ALS arrival and AAM, median (IQR), min	10.7 (6.8-15.9)	10.5 (7.0-15.0)	0.07	10.2 (6.2-15.5)	10.5 (7.1-14.7)	0.05
Departure from the scene						
Yes	1802 (96.6)	6454 (78.5)	0.57	1520 (49.2)	13 805 (49.5)	0.01
Interval between ALS arrival and departure from the scene, median (IQR), min	19.4 (14.6-25.2)	24.8 (19.4-31.7)	0.62	20.8 (15.1-26.7)	25.5 (19.5-32.6)	0.50

Abbreviations: AAM, advanced airway management; ALS, advanced life support; CPR, cardiopulmonary resuscitation; EMS, emergency medical services; IQR, interquartile range; NA, not available; PEA, pulseless electrical activity.

^a^ Data are given as No. (%) unless otherwise indicated.

^b^ Races that constituted the non-White category could not be ascertained from the deidentified patient-level data.

Using the time-dependent propensity scores, we performed 1:1 matching with replacement. Each patient receiving epinephrine at any given minute after EMS arrival was sequentially matched to a patient who was at risk of receiving epinephrine within the same minute to estimate the mean treatment effect (risk-set matching). At-risk patients included those who received epinephrine after the matching and those who never received epinephrine, because matching should be independent of future events.^11,12,13,14,15,21,22^ At-risk patients could have been subsequently matched multiple times as at-risk patients or as patients receiving epinephrine (only if the patients received epinephrine) until receiving epinephrine (matching with replacement).^12,13,15^ Matching with replacement was used to decrease bias by reducing the number of unmatched, exposed patients.^25^ Without replacement, the number of at-risk patients would have decreased as the matching progressed from time 0, and the number of unmatched patients who received epinephrine would have increased.^12,15,25^ We set the caliper width for the nearest-neighbor matching at 0.2 SD of the propensity scores in the logit scale.^25,26^ To assess the performance of the risk-set matching, we calculated the standardized difference for each covariate. We considered a standardized difference of less than 0.25 to be a well-matched balance.^25^

To ascertain whether there was an association between epinephrine administration and each outcome, we fitted a log link function in generalized estimating equations (GEEs) to estimate risk ratios (RRs) with 95% CIs compared with being at risk of receiving epinephrine (analyses without timing variable).^27^ We used GEEs to address potential within-pair correlation of risk-set matching. We used frequency weighting adjustment because some patients in the at-risk group could not be independent because of the matching with replacement.^25^

To evaluate the timing of epinephrine administration, we fitted 2 models with log link function in GEEs (analyses with timing variables) with frequency weighting adjustment. One model treated the timing of epinephrine as a categorical variable by 5-minute intervals. The other model treated timing of epinephrine administration as a continuous variable. In the model with the continuous variable, we included an interaction term between epinephrine administration and time to matching (ie, time from EMS arrival to the time of matching) and estimated the RRs of epinephrine at each minute, assuming a linear association between each outcome and the timing of epinephrine administration. Additional details are provided in the eMethods in the Supplement. When the *P* value for the interaction term was significant (*P* < .05), we considered the timing of epinephrine administration to be associated with the outcome. We calculated the change in RRs with 95% CIs per minute.

In addition, we conducted 3 sensitivity analyses. First, we performed the risk-set matching without replacement and repeated the same analysis except that we did not use frequency weighting adjustment because at-risk patients were independent. Second, we excluded those who had ROSC or TOR within 5 minutes after EMS arrival because these patients were successfully resuscitated or died before epinephrine could have been feasibly administered. We repeated the same time-dependent propensity score and risk-set matching analysis (matching with replacement). Third, we included only patients with bystander-witnessed arrest and repeated the time-dependent propensity score and risk-set matching analysis (matching with replacement). All tests were 2-sided; we regarded *P* < .05 as statistically significant. Data analysis was conducted from May 2019 to April 2021. All statistical analyses were performed with R software, version 3.5.1 (R Foundation for Statistical Computing). We reported statistical codes of time-dependent propensity score and risk-set matching in the eMethods in the Supplement.

## Results

A total of 41 079 adults with a median (interquartile range [IQR]) age of 67 (55-79) years (26 579 men [64.7%] and 14 481 women [35.3%]; 19 patients were missing information on sex), including 10 088 (24.6%) with shockable and 30 991 (75.4%) with nonshockable initial cardiac rhythms, were eligible for inclusion in the analyses (eFigure 1 in the Supplement). Functional outcome data were missing in 573 individuals (5.7%) with shockable and 417 (1.3%) with nonshockable rhythms.

Table 1 describes participants’ characteristics. Individuals with OHCA who received epinephrine included 8223 (81.5%) with a shockable cardiac rhythm and 27 901 (90.0%) with a nonshockable rhythm. The median (IQR) intervals between EMS arrival and epinephrine administration were 7.3 (5.3-10.0) minutes in those with shockable rhythms and 8.1 (6.0-11.0) minutes in those with nonshockable rhythms.

Using risk-set matching, 8213 patients with shockable and 27 882 with nonshockable initial cardiac rhythms who received epinephrine were matched with patients at risk of receiving epinephrine (Table 2). Among those matched as at-risk patients, 6626 (80.7%) in the shockable and 23 729 (85.1%) in the nonshockable rhythm cohorts received epinephrine after the matching. In both cohorts, standardized differences were within 0.25 for all variables, indicating a good postmatching balance. For shockable rhythms, median (IQR) intervals between arrival of ALS-trained EMS personnel and epinephrine administration were 7.0 (5.0-9.0) minutes for the epinephrine group and 10.0 (8.0-14.0) minutes for the at-risk group. For nonshockable rhythms, median (IQR) intervals between ALS arrival and epinephrine administration were 8.0 (6.0-11.0) minutes for the epinephrine group and 12.0 (9.0-15.0) minutes for the at-risk group.

**Table 2.** Characteristics and Covariates of Adults With Out-of-Hospital Cardiac Arrest With Epinephrine and at Risk of Receiving Epinephrine in the Time-Dependent Propensity Score–Matched Cohort^a^

Characteristic or Covariate	Shockable cardiac rhythms			Nonshockable cardiac rhythms		
	At risk of receiving epinephrine (n = 8213)	Epinephrine (n = 8213)	Standardized difference	At risk of receiving epinephrine (n = 27 882)	Epinephrine (n = 27 882)	Standardized difference
Age, median (IQR), y	65 (55-76)	65 (55-76)	0.04	68 (55-80)	68 (55-80)	0.01
Sex						
Male	6324 (77.0)	6409 (78.0)	0.02	16 903 (60.6)	17 098 (61.3)	0.02
Female	1889 (23.0)	1800 (21.9)		10 971 (39.3)	10 772 (38.6)	
Unknown	0	4 (0.0)		8 (0.0)	12 (0.0)	
Race						
White	1944 (23.7)	2084 (25.4)	0.04	6734 (24.2)	6837 (24.5)	0.01
Non-White^b^	6269 (76.3)	6129 (74.6)		21 148 (75.8)	21 045 (75.5)	
Cause						
Cardiac	8047 (98.0)	8067 (98.2)	0.02	25 922 (93.0)	25 803 (92.5)	0.02
Noncardiac	166 (2.0)	145 (1.8)		1960 (7.0)	2079 (7.5)	
Unknown	0	1 (0.0)		0		
Initial rhythm						
PEA (shockable)	NA	NA		7727 (27.7)	8247 (29.6)	0.04
Asystole (nonshockable)	NA	NA		20 155 (72.3)	19 635 (70.4)	
Location of cardiac arrest						
Street/highway	771 (9.4)	747 (9.1)	0.07	747 (2.7)	792 (2.8)	0.03
Public building	165 (2.0)	169 (2.1)		172 (0.6)	177 (0.6)	
Place of recreation	283 (3.4)	342 (4.2)		354 (1.3)	320 (1.1)	
Industrial place	172 (2.1)	178 (2.2)		129 (0.5)	133 (0.5)	
Home	5400 (65.7)	5314 (64.7)		21 032 (75.4)	21 100 (75.7)	
Farm/ranch	10 (0.1)	6 (0.1)		33 (0.1)	26 (0.1)	
Health care facility	121 (1.5)	132 (1.6)		524 (1.9)	566 (2.0)	
Residential institution	358 (4.4)	300 (3.7)		3604 (12.9)	3436 (12.3)	
Other public property	872 (10.6)	956 (11.6)		1123 (4.0)	1197 (4.3)	
Other nonpublic property	37 (0.5)	49 (0.6)		104 (0.4)	83 (0.3)	
Unknown	24 (0.3)	20 (0.2)		60 (0.2)	52 (0.2)	
Witnessed collapse						
Bystander	5184 (63.1)	5338 (65.0)	0.04	9190 (33.0)	9429 (33.8)	0.02
None	2852 (34.7)	2689 (32.7)		17 871 (64.1)	17 678 (63.4)	
Unknown	177 (2.2)	186 (2.3)		821 (2.9)	775 (2.8)	
Layperson CPR						
Yes	4344 (52.9)	4680 (57.0)	0.08	12 909 (46.3)	13 168 (47.2)	0.02
No	3727 (45.4)	3389 (41.3)		14 358 (51.5)	14 114 (50.6)	
Unknown	142 (1.7)	144 (1.8)		615 (2.2)	600 (2.2)	
Shock delivery before arrival of ALS-trained EMS personnel						
Yes	2028 (24.7)	2381 (29.0)	0.10	312 (1.1)	304 (1.1)	0.01
No	6185 (75.3)	5832 (71.0)		27 570 (98.9)	27 578 (98.9)	
EMS response time (interval between 9-1-1 call and first EMS arrival), median (IQR), min	5.5 (4.3-7.0)	5.5 (4.3-7.0)	0.03	5.4 (4.2-7.0)	5.4 (4.2-7.0)	0.01
Shock delivery after ALS arrival						
Yes	6114 (74.4)	5667 (69.0)	0.12	4049 (14.5)	4486 (16.1)	0.04
Interval between ALS arrival and shock delivery, median (IQR), min	4.2 (2.7-6.0)	4.0 (2.5-5.9)	0.05	15.3 (8.5-22.6)	13.5 (8.5-19.9)	0.14
Advanced airway management			0.11			
Yes	6303 (76.7)	6673 (81.2)	0.11	20 716 (74.3)	21 883 (78.5)	0.10
Interval between ALS arrival and AAM, median (IQR), min	11.0 (7.0-16.0)	10.5 (7.0-15.0)	0.07	11.0 (7.0-16.0)	10.5 (7.1-14.7)	0.11
Departure from the scene						
Yes	6592 (80.3)	6446 (78.5)	0.04	14 270 (51.2)	13 789 (49.5)	0.04
Interval between ALS arrival and departure from the scene, median (IQR), min	24.8 (19.1-32.0)	24.8 (19.4-31.7)	0.001	25.6 (19.0-33.5)	25.4 (19.5-32.6)	0.03

Abbreviations: AAM, advanced airway management; ALS, advanced life support; CPR, cardiopulmonary resuscitation; EMS, emergency medical services; IQR, interquartile range; NA, not available; PEA, pulseless electrical activity.

^a^ Data are given as No. (%) unless otherwise indicated.

^b^ Races that constituted the non-White category could not be ascertained from the deidentified patient-level data.

### Analyses Without Timing Variable

For shockable cardiac rhythms, receipt of epinephrine was not associated with survival to hospital discharge compared with being at risk of receiving epinephrine (RR, 0.96; 95% CI, 0.89-1.03) (Table 3). Although receipt of epinephrine was not associated with favorable functional outcome (RR, 0.95; 95% CI, 0.87-1.04), epinephrine was associated with prehospital ROSC (RR, 1.16; 95% CI, 1.12-1.21).

**Table 3.** Outcomes in Time-Dependent Propensity Score–Matched Cohort

Outcomes	No. of patients with outcome/total No. of patients (%)		Risk ratio (95% CI)
	At risk of receiving epinephrine	Epinephrine	
**Shockable cardiac rhythms**			
Analyses without timing variable			
Survival to hospital discharge	1721/8213 (21.0)	1548/8213 (18.8)	0.96 (0.89-1.03)
Favorable functional outcome at hospital discharge	1364/8213 (16.6)	1204/8213 (14.7)	0.95 (0.87-1.04)
Prehospital ROSC	3613/8213 (44.0)	3971/8213 (48.4)	1.16 (1.12-1.21)
Analyses with categorized timing variable			
Survival to hospital discharge, min after EMS arrival			
0-5	452/1637 (27.6)	454/1637 (27.7)	1.12 (0.99-1.26)
5-10	944/4540 (20.8)	877/4540 (19.3)	1.07 (0.97-1.17)
10-15	252/1549 (16.3)	185/1549 (11.9)	0.80 (0.66-0.98)
15-20	56/369 (15.2)	27/369 (7.3)	0.55 (0.33-0.89)
>20	17/118 (14.4)	5/118 (4.2)	0.13 (0.05-0.37)
Favorable functional outcome at hospital discharge, min after EMS arrival			
0-5	384/1637 (23.5)	364/1637 (22.2)	1.07 (0.93-1.23)
5-10	735/4540 (16.2)	688/4540 (15.2)	1.10 (0.99-1.23)
10-15	188/1549 (12.1)	131/1549 (8.5)	0.76 (0.60-0.97)
15-20	41/369 (11.1)	18/369 (4.9)	0.54 (0.30-0.99)
>20	16/118 (13.6)	3/118 (2.5)	0.07 (0.02-0.30)
Prehospital ROSC, min after EMS arrival			
0-5	912/1637 (55.7)	987/1637 (60.3)	1.16 (1.09-1.24)
5-10	2130/4540 (46.9)	2290/4540 (50.4)	1.16 (1.11-1.22)
10-15	493/1549 (31.8)	580/1549 (37.4)	1.28 (1.15-1.43)
15-20	67/369 (18.2)	95/369 (25.7)	1.57 (1.13-2.19)
>20	11/118 (9.3)	19/118 (16.1)	1.50 (0.67-3.38)
**Nonshockable cardiac rhythms**			
Analyses without timing variable			
Survival to hospital discharge	635/27 882 (2.3)	611/27 882 (2.2)	1.01 (0.88-1.15)
Favorable functional outcome at hospital discharge	333/27 882 (1.2)	262/27 882 (0.9)	0.84 (0.68-1.02)
Prehospital ROSC	6020/27 882 (21.6)	7932/27 882 (28.4)	1.35 (1.31-1.40)
Analyses with categorized timing variable			
Survival to hospital discharge, min after ALS arrival			
0-5	113/3878 (2.9)	108/3878 (2.8)	1.28 (0.95-1.72)
5-10	355/14 758 (2.4)	361/14 758 (2.4)	1.14 (0.96-1.34)
10-15	118/6649 (1.8)	117/6649 (1.8)	1.01 (0.75-1.35)
15-20	33/1871 (1.8)	21/1871 (1.1)	0.60 (0.31-1.15)
>20	16/726 (2.2)	4/726 (0.6)	0.36 (0.11-1.23)
Favorable functional outcome at hospital discharge, min after ALS arrival			
0-5	58/3878 (1.5)	51/3878 (1.3)	1.26 (0.81-1.95)
5-10	178/14 758 (1.2)	150/14 758 (1.0)	0.96 (0.74-1.24)
10-15	65/6649 (1.0)	50/6649 (0.8)	0.82 (0.52-1.28)
15-20	19/1871 (1.0)	10/1871 (0.5)	0.45 (0.18-1.11)
>20	12/726 (1.7)	2/726 (0.3)	0.19 (0.02-1.52)
Prehospital ROSC, min after ALS arrival			
0-5	1016/3878 (26.2)	1312/3878 (33.8)	1.42 (1.32-1.53)
5-10	3582/14 758 (24.3)	4533/14 758 (30.7)	1.34 (1.28-1.39)
10-15	1178/6649 (17.7)	1647/6649 (24.8)	1.42 (1.32-1.53)
15-20	201/1871 (10.7)	348/1871 (18.6)	1.70 (1.42-2.03)
>20	43/726 (5.9)	92/726 (12.7)	2.14 (1.45-3.15)

Abbreviations: ALS, advanced life support; EMS, emergency medical services; ROSC, return of spontaneous circulation.

In the nonshockable cardiac rhythm cohort, survival to hospital discharge (RR, 1.01; 95% CI, 0.88-1.15) and favorable functional outcome (RR, 0.84; 95% CI, 0.68-1.02) did not differ between the epinephrine and at-risk groups. However, receipt of epinephrine was associated with prehospital ROSC (RR, 1.35; 95% CI, 1.31-1.40).

### Analyses With Timing Variables

**Figure 1.**
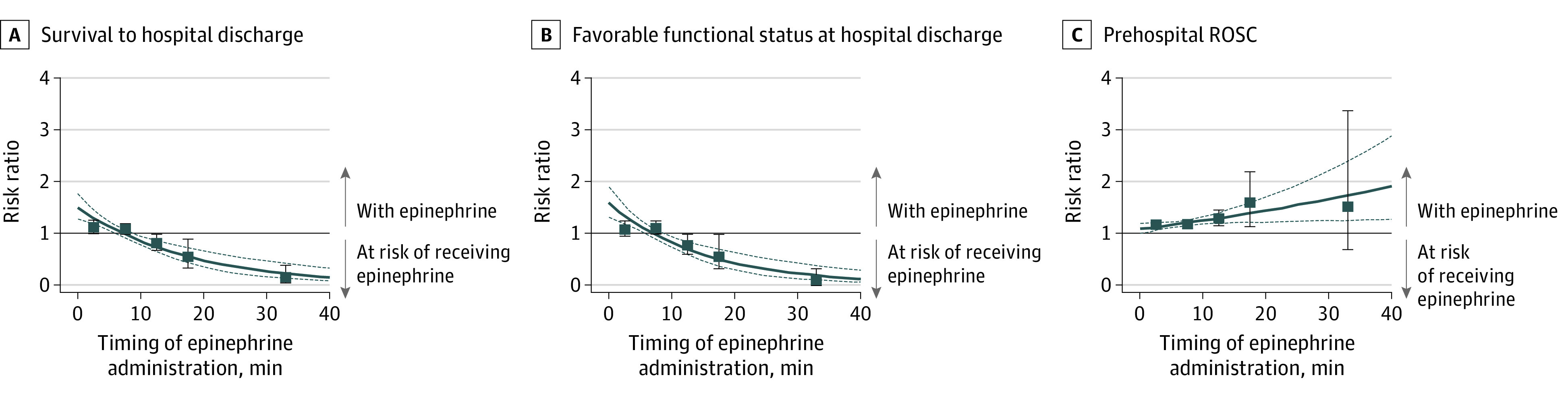
Survival to Hospital Discharge, Favorable Functional Outcome at Hospital Discharge, and Prehospital Return of Spontaneous Circulation (ROSC) Stratified by Timing of Epinephrine Administration in Patients With Out-of-Hospital Cardiac Arrest and Initial Shockable Cardiac Rhythms Figure shows the risk ratio (RR) point estimates (squares) with the 95% CIs (upper and lower bounds indicated by the blue dashed lines) for administration of epinephrine after arrival of emergency medical services personnel at the scene associated with survival to hospital discharge (A), favorable functional status at discharge (B), and ROSC (C). Timing of epinephrine administration was treated as a continuous variable. A, The RR per minute decreased 5.5% (95% CI, 3.4%-7.5%; *P* < .001 for the interaction). B, The RR per minute decreased 6.4% (95% CI, 3.8%-8.9%; *P* < .001 for the interaction). C, The RR per minute increased 1.4% (95% CI, 0.2%-2.7%; *P* = .02 for the interaction). The solid line represents the outcome. Risk ratios greater than 1.00 (horizontal line) favored receiving epinephrine; those less than 1.00, not receiving epinephrine. The error bars indicate 95% CIs.

Figure 1 and Table 3 show the RRs of epinephrine administration associated with outcomes stratified according to the timing of epinephrine administration for shockable cardiac rhythms. The RR point estimates in the time-dependent propensity score–matched cohorts for the analysis of epinephrine administration and survival to hospital discharge were 1.12 (95% CI, 0.99-1.26) for 0-5 minutes, 1.07 (95% CI, 0.97-1.17) for 5 to 10 minutes, 0.80 (95% CI, 0.66-0.98) for 10 to 15 minutes, 0.55 (95% CI, 0.33-0.89) for 15 to 20 minutes, and 0.13 (95% CI, 0.05-0.37) for more than 20 minutes after ALS arrival (Figure 1A and Table 3). Treating the timing of epinephrine as a continuous variable, RRs decreased 5.5% (95% CI, 3.4%-7.5%; *P* < .001 for the interaction) for survival to hospital discharge (Figure 1A) and 6.4% (95% CI, 3.8%-8.9%; *P* < .001 for the interaction) for functional outcome (Figure 1B) per minute after EMS arrival. In contrast, the RR for prehospital ROSC increased 1.4% per minute after EMS arrival (95% CI, 0.2%-2.7%, *P* = .02 for the interaction) (Figure 1C).

**Figure 2.**
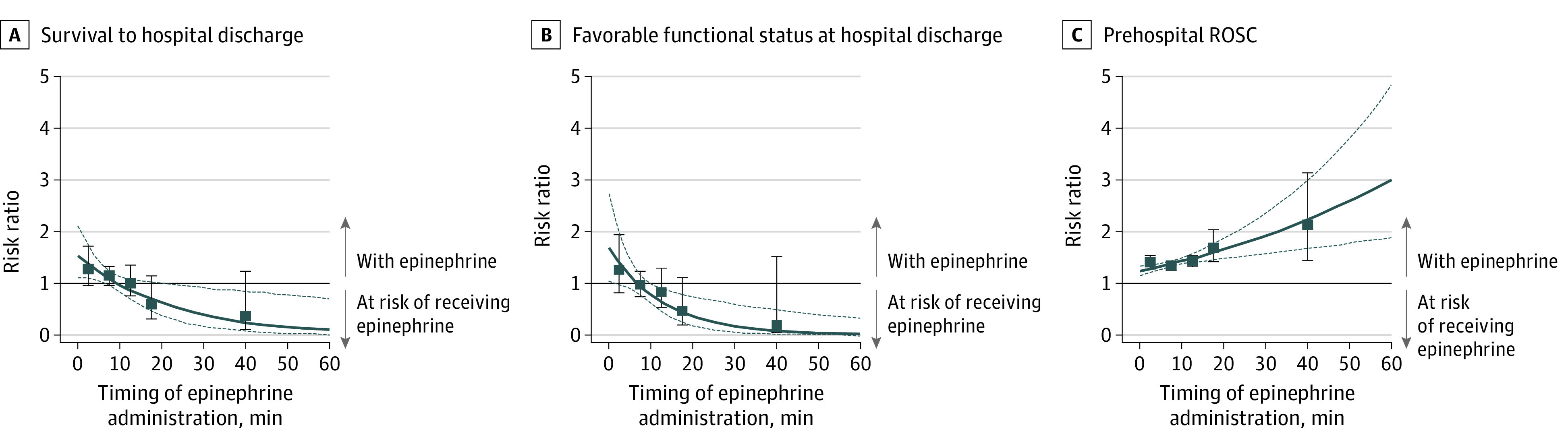
Survival to Hospital Discharge, Favorable Functional Outcome at Hospital Discharge, and Prehospital Return of Spontaneous Circulation (ROSC) Stratified by the Timing of Epinephrine Administration in Patients With Out-of-Hospital Cardiac Arrest and Initial Nonshockable Cardiac Rhythms Figure shows the risk ratio point estimates (squares) with the 95% CIs (upper and lower bounds indicated by the blue dashed lines) associated with survival to hospital discharge (A), favorable functional status at discharge (B), and ROSC (C). Timing of epinephrine administration was treated as a continuous variable. A, The RR per minute decreased 4.4% (95% CI, 0.8%-7.9%; *P* = .02 for the interaction). B, The RR per minute decreased 7.1% (95% CI, 1.7%-12.3%; *P* = .01 for the interaction). C, The RR per minute increased 1.5% (95% CI, 0.6%-2.4%; *P* = .001 for the interaction). The solid line represents the outcome. Risk ratios greater than 1.00 (horizontal line) favored receiving epinephrine; those less than 1.00, not receiving epinephrine. The error bars indicate 95% CIs.

Figure 2 and Table 3 show the RRs of epinephrine administration associated with outcomes stratified according to the timing of epinephrine administration for nonshockable cardiac rhythms. The point estimates for administration of epinephrine and survival to hospital discharge were 1.28 (95% CI, 0.95-1.72) for 0 to 5 minutes, 1.14 (95% CI, 0.96-1.34) for 5 to 10 minutes, 1.01 (95% CI, 0.75-1.35) for 10 to 15 minutes, 0.60 (95% CI, 0.31-1.15) for 15 to 20 minutes, and 0.36 (95% CI, 0.11-1.23) for more than 20 minutes (Figure 2A and Table 3). Treating the timing of epinephrine as a continuous variable, RRs for survival to hospital discharge decreased 4.4% per minute (95% CI, 0.8%-7.9%; *P* = .02 for the interaction) (Figure 2A) and 7.1% per minute (95% CI, 1.7%-12.3%; *P* = .01 for the interaction) for functional outcome (Figure 2B). The RR for prehospital ROSC increased 1.5% per minute after ALS-trained EMS personnel arrival (95% CI, 0.6%-2.4%, *P* = .001 for the interaction).

### Sensitivity Analyses

Characteristics of the baseline (eTables 3 and 6 in the Supplement) and matched cohorts (eTables 1, 4, and 7 in the Supplement) had good postmatching balance. Risk ratios of epinephrine administration associated with outcomes in the matched cohorts were similar to the results of the primary analysis (eTables 2, 5, and 8 in the Supplement). eFigures 2-7 in the Supplement show the RR point estimates for receipt of epinephrine associated with outcomes stratified according to the timing of epinephrine administration. For shockable cardiac rhythms, the timing of epinephrine administration (*P* values for the interaction term) was associated with survival to hospital discharge and favorable functional outcome in all sensitivity analyses. In contrast, for nonshockable rhythms, the timing of epinephrine administration was not associated with survival to hospital discharge or favorable functional outcome in the analyses with risk-set matching without replacement (eFigure 3A and B in the Supplement) and with bystander-witnessed OHCA (eFigure 7A and B in the Supplement). The sensitivity analysis excluding those who had ROSC or TOR within 5 minutes after ALS-trained EMS personnel arrival showed findings similar to the primary analysis in shockable and nonshockable rhythms (eFigures 4 and 5 in the Supplement).

## Discussion

In this cohort study with a time-dependent propensity score and risk-set matching analysis performed using data from a large OHCA registry with 10 sites in North America, the association of receipt of epinephrine with survival to hospital discharge and favorable functional outcome differed based on the timing of the administration in adults with OHCA and initial cardiac rhythms that were shockable or nonshockable. The findings were consistent among 3 sensitivity analyses for shockable rhythms; however, for nonshockable rhythms, the timing of epinephrine was not associated with survival to hospital discharge and favorable functional outcome in analyses of matching without replacement and with bystander-witnessed OHCAs, which may be explained by the smaller sample size (29 506 patients in the analysis without replacement and 18 836 patients in the analysis of bystander-witnessed OHCAs) and limited outcome events in these analyses.

### Comparison With Previous Studies

Previous studies have reported inconsistent findings about the timing of epinephrine administration in patients with OHCA. A retrospective observational study in Michigan found that early epinephrine administration (time from 9-1-1 call to epinephrine administration, ≤10 minutes) was not associated with survival to hospital discharge (odds ratio [OR], 0.91; 95% CI, 0.35-2.37) for adults with OHCA compared with late epinephrine administration (time from 9-1-1 call to epinephrine administration, >10 minutes).^28^ A secondary analysis of a clinical trial in the UK that included 4810 patients with OHCA found that 30-day survival (interaction OR, 0.98; 95% CI, 0.94-1.03) and favorable functional outcomes at hospital discharge (interaction OR, 0.98; 95% CI, 0.93-1.03) were not substantively different over time between the epinephrine and placebo groups, suggesting that timing of epinephrine administration is not an effect modifier on survival and functional outcome.^29^ In contrast, a secondary analysis of the ROC registry reported that each additional minute of time from EMS arrival to epinephrine administration was associated with a 4% decrease in the odds of survival to hospital discharge (OR, 0.96; 95% CI, 0.95-0.98) for patients with nonshockable cardiac rhythms.^30^ In a 2019 systematic review, the authors recognized that all included studies investigating the timing of epinephrine administration had a critical risk of bias attributable to confounding and/or selection bias.^7^ The high degree of heterogeneity across the studies and the serious critical risk of bias precluded any meaningful assessment of the optimal timing of epinephrine administration.^7^

### Implications

The latest resuscitation guidelines and international recommendations emphasize early epinephrine administration for OHCA.^5,6^ Findings of the present study support earlier epinephrine administration for OHCA with shockable and nonshockable cardiac rhythms and provide further evidence to complement these guidelines and recommendations. Another implication is that later epinephrine was found to be associated with ROSC but inversely associated with survival to hospital discharge and favorable functional outcomes. This discordance may suggest that later epinephrine administration might not be beneficial for survival to hospital discharge and functional recovery. The reasons for this discordance are unclear, but it is possible that longer resuscitation time may be associated with poor outcome, and epinephrine is the only intervention associated with increased odds of ROSC in patients with OHCA.

### Strengths and Limitations

This study has strengths. First, we addressed resuscitation time bias and time-varying confounders. Although the secondary analysis of a clinical trial in the UK also addressed resuscitation time bias given its randomized double-blind design,^29^ the difference in the results of that study and the present study may be explained by the differences in time to epinephrine administration (median intervals between EMS arrival and epinephrine administration were 14.8 minutes in the epinephrine group and 14.5 minutes in the placebo group in the UK study) and sample size. Second, RRs in the present study should be interpreted as the ratio of the risk of outcomes with epinephrine at any given minute vs the risk of outcomes without epinephrine at the same minute. This interpretation is clinically relevant for deciding whether a patient should receive epinephrine now.

This study also has limitations. First, the timing of epinephrine administration may be a surrogate of EMS performance (ie, high-performing EMS personnel may administer epinephrine early). Because information on EMS systems was unavailable, we were unable to adjust for the clustering of patients within EMS systems. Similarly, we were unable to adjust for unmeasured confounders, such as patient comorbidity, postresuscitation practice,^31^ and neighborhood factors.^32^ Second, we cannot eliminate confounding by indication (ie, EMS personnel may not have administered epinephrine in patients who were expected to have early ROSC without epinephrine or in patients who had early TOR due to futility).^33^ To account for this, we conducted a sensitivity analysis excluding those who had ROSC or TOR within 5 minutes after ALS arrival and observed consistent results. However, residual confounding by indication may still exist. For example, we defined cases as patients who were successfully administered epinephrine, whereas patients who had delayed epinephrine administration because of difficulty in establishing vascular access could have been matched as a control.^34^ Third, given the observational design of the present study, we could not demonstrate causation. A clinical trial comparing early vs late epinephrine could assess for a causal association between early epinephrine administration and patient outcomes. However, given the current evidence about the survival benefit of epinephrine,^3^ such a trial would not be ethically feasible. Fourth, the findings of the present study may not be generalizable to other EMS systems.

## Conclusions

In this cohort study of more than 40 000 adults with OHCAs in North America, for both initial shockable and nonshockable cardiac rhythms, we found that the associations of epinephrine administration with survival to hospital discharge and favorable functional status at hospital discharge differed on the basis of the timing of administration, and risk ratios for the association between receipt of epinephrine and patient outcomes decreased as administration of epinephrine was delayed.

## Notes

Supplement.
eMethods.

eReferences.
**eFigure 1.** Patient Flow **eFigure 2.** Association of Epinephrine Administration With Survival to Hospital Discharge (A), Favorable Functional Outcome at Hospital Discharge (B), and Prehospital ROSC (C) by the Timing of the Administration for Patients With Shockable OHCA (Matching Without Replacement) **eFigure 3.** Association of Epinephrine Administration With Survival to Hospital Discharge (A), Favorable Functional Outcome at Hospital Discharge (B), and Prehospital ROSC (C) by the Timing of the Administration for Patients With Nonshockable OHCA (Matching Without Replacement) **eFigure 4.** Association of Epinephrine Administration With Survival to Hospital Discharge (A), Favorable Functional Outcome at Hospital Discharge (B), and Prehospital ROSC (C) by the Timing of the Administration for Patients With Shockable OHCA Excluding Those Who Had ROSC or TOR Within 5 Minutes After ALS EMS Arrival on the Scene **eFigure 5.** Association of Epinephrine Administration With Survival to Hospital Discharge (A), Favorable Functional Outcome at Hospital Discharge (B), and Prehospital ROSC (C) by the Timing of the Administration for Patients With Nonshockable OHCA Excluding Those Who Had ROSC or TOR Within 5 Minutes After ALS EMS Arrival on the Scene **eFigure 6.** Association of Epinephrine Administration With Survival to Hospital Discharge (A), Favorable Functional Outcome at Hospital Discharge (B), and Prehospital ROSC (C) by the Timing of the Administration for Patients With Bystander Witnessed Shockable OHCA **eFigure 7.** Association of Epinephrine Administration With Survival to Hospital Discharge (A), Favorable Functional Outcome at Hospital Discharge (B), and Prehospital ROSC (C) for Patients With Bystander Witnessed Nonshockable OHCA **eTable 1.** Characteristics of Adult Patients With Out-of-Hospital Cardiac Arrest With Epinephrine and at Risk of Receiving Epinephrine in Time-Dependent Propensity Score Matched Cohort (Matching Without Replacement) **eTable 2.** Outcomes in Time-Dependent Propensity Score Matched Cohort (Matching Without Replacement) **eTable 3.** Characteristics of Adult Patients With Out-of-Hospital Cardiac Arrest With and Without Epinephrine in Original Cohort, Excluding Those Who Had ROSC or TOR Within 5 Minutes After ALS EMS Arrival **eTable 4.** Characteristics of Adult Patients With Out-of-Hospital Cardiac Arrest With Epinephrine and at Risk of Receiving Epinephrine in Time-Dependent Propensity Score Matched Cohort, Excluding Those Who Had ROSC or TOR Within 5 Minutes After ALS EMS Arrival **eTable 5.** Outcomes in Time-Dependent Propensity Score Matched Cohort, Excluding Those Who Had ROSC or TOR Within 5 Minutes After ALS EMS Arrival **eTable 6.** Characteristics of Adult Patients With Bystander Witnessed Out-of-Hospital Cardiac Arrest With and Without Epinephrine **eTable 7.** Characteristics of Adult Patients With Bystander Witnessed Out-of-Hospital Cardiac Arrest With Epinephrine and at Risk of Receiving Epinephrine in Time-Dependent Propensity Score Matched Cohort **eTable 8.** Outcomes in Time-Dependent Propensity Score Matched Cohort of Bystander Witnessed Out-of-Hospital Cardiac Arrest
