# Supplementary material for: Association of Timing of Epinephrine Administration With Outcomes in Adults With Out-of-Hospital Cardiac Arrest
Source: JAMA Netw Open. 2021 Aug 10;4(8):e2120176. doi: 10.1001/jamanetworkopen.2021.20176 (PMC8356068; doi:10.1001/jamanetworkopen.2021.20176)
Supplement: Supplement. — eMethods. eReferences. eFigure 1. Patient Flow eFigure 2. Association of Epinephrine Administration With Survival to Hospital Discharge (A), Favorable Functional Outcome at Hospital Discharge (B), and Prehospital ROSC (C) by the Timing of the Administration for Patients With Shockable OHCA (Matching Without Replacement) eFigure 3. Association of Epinephrine Administration With Survival to Hospital Discharge (A), Favorable Functional Outcome at Hospital Discharge (B), and Prehospital ROSC (C) by the Timing of the Administration for Patients With Nonshockable OHCA (Matching Without Replacement) eFigure 4. Association of Epinephrine Administration With Survival to Hospital Discharge (A), Favorable Functional Outcome at Hospital Discharge (B), and Prehospital ROSC (C) by the Timing of the Administration for Patients With Shockable OHCA Excluding Those Who Had ROSC or TOR Within 5 Minutes After ALS EMS Arrival on the Scene eFigure 5. Association of Epinephrine Administration With Survival to Hospital Discharge (A), Favorable Functional Outcome at Hospital Discharge (B), and Prehospital ROSC (C) by the Timing of the Administration for Patients With Nonshockable OHCA Excluding Those Who Had ROSC or TOR Within 5 Minutes After ALS EMS Arrival on the Scene eFigure 6. Association of Epinephrine Administration With Survival to Hospital Discharge (A), Favorable Functional Outcome at Hospital Discharge (B), and Prehospital ROSC (C) by the Timing of the Administration for Patients With Bystander Witnessed Shockable OHCA eFigure 7. Association of Epinephrine Administration With Survival to Hospital Discharge (A), Favorable Functional Outcome at Hospital Discharge (B), and Prehospital ROSC (C) for Patients With Bystander Witnessed Nonshockable OHCA eTable 1. Characteristics of Adult Patients With Out-of-Hospital Cardiac Arrest With Epinephrine and at Risk of Receiving Epinephrine in Time-Dependent Propensity Score Matched Cohort (Matching Without Replacement) eTable 2. Outcomes in T [file jamanetwopen-e2120176-s001.pdf]

**Supplemental Online Content**

Okubo M, Komukai S, Callaway CW, Izawa J. Association of Timing of Epinephrine Administration With Outcomes in Adults With Out-of-Hospital Cardiac Arrest. *JAMA Netw Open.* 2021;4(8):e2120176. doi:10.1001/jamanetworkopen.2021.20176

**eMethods.**

**eReferences.**

**eFigure 1.** Patient Flow

**eFigure 2.** Association of Epinephrine Administration With Survival to Hospital Discharge (A), Favorable Functional Outcome at Hospital Discharge (B), and Prehospital ROSC (C) by the Timing of the Administration for Patients With Shockable OHCA (Matching Without Replacement)

**eFigure 3.** Association of Epinephrine Administration With Survival to Hospital Discharge (A), Favorable Functional Outcome at Hospital Discharge (B), and Prehospital ROSC (C) by the Timing of the Administration for Patients With Nonshockable OHCA (Matching Without Replacement)

**eFigure 4.** Association of Epinephrine Administration With Survival to Hospital Discharge (A), Favorable Functional Outcome at Hospital Discharge (B), and Prehospital ROSC (C) by the Timing of the Administration for Patients With Shockable OHCA Excluding Those Who Had ROSC or TOR Within 5 Minutes After ALS EMS Arrival on the Scene

**eFigure 5.** Association of Epinephrine Administration With Survival to Hospital Discharge (A), Favorable Functional Outcome at Hospital Discharge (B), and Prehospital ROSC (C) by the Timing of the Administration for Patients With Nonshockable OHCA Excluding Those Who Had ROSC or TOR Within 5 Minutes After ALS EMS Arrival on the Scene

**eFigure 6.** Association of Epinephrine Administration With Survival to Hospital Discharge (A), Favorable Functional Outcome at Hospital Discharge (B), and Prehospital ROSC (C) by the Timing of the Administration for Patients With Bystander Witnessed Shockable OHCA

**eFigure 7.** Association of Epinephrine Administration With Survival to Hospital Discharge (A), Favorable Functional Outcome at Hospital Discharge (B), and Prehospital ROSC (C) for Patients With Bystander Witnessed Nonshockable OHCA

**eTable 1.** Characteristics of Adult Patients With Out-of-Hospital Cardiac Arrest With Epinephrine and at Risk of Receiving Epinephrine in Time-Dependent Propensity Score Matched Cohort (Matching Without Replacement)

**eTable 2.** Outcomes in Time-Dependent Propensity Score Matched Cohort (Matching Without Replacement)

**eTable 3.** Characteristics of Adult Patients With Out-of-Hospital Cardiac Arrest With and Without Epinephrine in Original Cohort, Excluding Those Who Had ROSC or TOR Within 5 Minutes After ALS EMS Arrival

**eTable 4.** Characteristics of Adult Patients With Out-of-Hospital Cardiac Arrest With Epinephrine and at Risk of Receiving Epinephrine in Time-Dependent Propensity Score Matched Cohort, Excluding Those Who Had ROSC or TOR Within 5 Minutes After ALS EMS Arrival

**eTable 5.** Outcomes in Time-Dependent Propensity Score Matched Cohort, Excluding Those Who Had ROSC or TOR Within 5 Minutes After ALS EMS Arrival

**eTable 6.** Characteristics of Adult Patients With Bystander Witnessed Out-of-Hospital Cardiac Arrest With and Without Epinephrine

**eTable 7.** Characteristics of Adult Patients With Bystander Witnessed Out-of-Hospital Cardiac Arrest With Epinephrine and at Risk of Receiving Epinephrine in Time-Dependent Propensity Score Matched Cohort

**eTable 8.** Outcomes in Time-Dependent Propensity Score Matched Cohort of Bystander Witnessed Out-of-Hospital Cardiac Arrest

This supplemental material has been provided by the authors to give readers additional information about their work.

© 2021 Okubo M et al. *JAMA Network Open.*

**eMethods.**

*Time-dependent propensity score*

We calculated propensity score as the time-varying probability of receiving epinephrine which was estimated from the hazard component at any given minutes after ALS arrival from Fine-Gray regression model with time-dependent covariates, time-independent covariates, competing risk events, and a censoring.[1-4] The time-dependent covariates were shock delivery (if a patient received shock), AAM (if a patient received AAM), and departure from the scene (if a patient was transported) after ALS arrival. The time-independent covariates were patient age, sex, race, location of arrest, etiology of arrest, witness status, layperson CPR, shock delivery before ALS arrival, and EMS response time. The time-dependent and time-independent covariates are presented in Table 1. We used spline functions (B-spline) for continuous variables (age and EMS response time). We chose these covariates a priori based on their association with survival from prior knowledge, biologic plausibility, and adequate ascertainment.[5-10] We included prehospital ROSC and TOR before epinephrine administration as competing risks in the model because (1) epinephrine administration never occurs after ROSC and TOR except cases with re-arrest after ROSC, (2) our interest was timing of epinephrine for initial arrest, and (3) ROSC and TOR are informative censorings. We modelled hospital arrival as a censoring because our main interest was the timing of the first prehospital epinephrine administration.

*Linear assumption in a model treating the timing of epinephrine as a continuous variable*

We assumed a linear relation between each outcome and the timing of epinephrine administration. To investigate the robustness of this linear assumption, we demonstrated the overlapping of RRs with 95% CIs in two models. We also explored a spline regression model assuming a nonlinear relationship between each outcome and the timing of epinephrine

© 2021 Okubo M et al. *JAMA Network Open.*

administration. The linear model showed smaller quasi-likelihood under the independence model criterion (QIC) for our primary outcome in both shockable and nonshockable cohorts, compared with the spline regression model. Since prior literature recommends using a GEE model with the smallest QIC, we chose the linear model.[11]

**References:**


1. Izawa J, Komukai S, Gibo K, et al. Pre-hospital advanced airway management for adults with out-of-hospital cardiac arrest: nationwide cohort study. *BMJ.* 2019;364:l430.
2. Matsuyama T, Komukai S, Izawa J, et al. Pre-Hospital Administration of Epinephrine in Pediatric Patients With Out-of-Hospital Cardiac Arrest. *J Am Coll Cardiol.* 2020;75(2):194-204.
3. Okubo M, Komukai S, Izawa J, et al. Prehospital advanced airway management for paediatric patients with out-of-hospital cardiac arrest: A nationwide cohort study. *Resuscitation.* 2019;145:175-184.
4. Beyersmann J, Schumacher M. Time-dependent covariates in the proportional subdistribution hazards model for competing risks. *Biostatistics.* 2008;9(4):765-776.
5. Kleinman ME, Brennan EE, Goldberger ZD, et al. Part 5: Adult Basic Life Support and Cardiopulmonary Resuscitation Quality: 2015 American Heart Association Guidelines Update for Cardiopulmonary Resuscitation and Emergency Cardiovascular Care. *Circulation.* 2015;132(18 Suppl 2):S414-435.
6. Link MS, Berkow LC, Kudenchuk PJ, et al. Part 7: Adult Advanced Cardiovascular Life Support: 2015 American Heart Association Guidelines Update for Cardiopulmonary Resuscitation and Emergency Cardiovascular Care. *Circulation.* 2015;132(18 Suppl 2):S444-464.
7. Panchal AR, Bartos JA, Cabanas JG, et al. Part 3: Adult Basic and Advanced Life Support: 2020 American Heart Association Guidelines for Cardiopulmonary Resuscitation and Emergency Cardiovascular Care. *Circulation.* 2020;142(16_suppl_2):S366-S468.
8. Panchal AR, Berg KM, Hirsch KG, et al. 2019 American Heart Association Focused Update on Advanced Cardiovascular Life Support: Use of Advanced Airways, Vasopressors, and Extracorporeal Cardiopulmonary Resuscitation During Cardiac Arrest: An Update to the American Heart Association Guidelines for Cardiopulmonary Resuscitation and Emergency Cardiovascular Care. *Circulation.* 2019;140(24):e881-e894.
9. Sasson C, Rogers MA, Dahl J, Kellermann AL. Predictors of survival from out-of-hospital cardiac arrest: a systematic review and meta-analysis. *Circ Cardiovasc Qual Outcomes.* 2010;3(1):63-81.
10. Soar J, Maconochie I, Wyckoff MH, et al. 2019 International Consensus on Cardiopulmonary Resuscitation and Emergency Cardiovascular Care Science With Treatment Recommendations: Summary From the Basic Life Support; Advanced Life


© 2021 Okubo M et al. *JAMA Network Open.*

Support; Pediatric Life Support; Neonatal Life Support; Education, Implementation, and Teams; and First Aid Task Forces. *Circulation.* 2019;140(24):e826-e880.
11. Pan W. Akaike's information criterion in generalized estimating equations. *Biometrics.* 2001;57:120-125.

© 2021 Okubo M et al. *JAMA Network Open.*

*Statistical codes of time-dependent propensity score and risk-set matching*

```
if(0){
	install.packages( "dplyr"	, repos = "http://cran.r-project.org/" )
	install.packages( "plyr"	, repos = "http://cran.r-project.org/" )
	install.packages( "tidyr"	, repos = "http://cran.r-project.org/" )
	install.packages( "Matching"	, repos = "http://cran.r-project.org/" )
}

library( dplyr )
library( plyr )
library( tidyr )
library( Matching )

#------------#
#--- Data ---#
#------------#

#--------------------------------------------------------------------------------------------#
# analysisdata: long data with time-dependent covariates
#
#  analysisdata has the following variables
#   treatment         : treatment indicator
#   time_to_treatment : duration from time of inclution to time of treatment
#   outcome           : outcome variable
#   PS                : time-dependent propensity score which is estimated by survival model
#   start             : the starting time for the interval in which the patient has that time-dependent covariates
#   stop              : the stopped time for the interval in which the patient has that time-dependent covariates
#
#  This data has some time-independent variables and time-dependent variables
#
# seed              : seed for random numbers
#--------------------------------------------------------------------------------------------#
#
# We set only analysisdata and seed.
#

#------------------------------------------------------------------#
#--- Sequential matching algorithm using time-dependent variable ---#
#------------------------------------------------------------------#
library( Matching )

j.ind              <- sort( unique( analysisdata$time_to_treatment ) )
```

© 2021 Okubo M et al. *JAMA Network Open.*

```
matchinglist              <- list()
exclude.ids               <- c()
set.seed( seed )

for( j in j.ind ){   # Interval time is one minute
        subdata                  <- analysisdata

        #--- treatment data ---#
        subind1                  <- ( subdata$treatment == 1 & subdata$time_to_treatment == j )
        treatmentcandidates      <- subdata[ subind1, ]
        if( nrow( treatmentcandidates ) == 0 ){
                matchinglist[[ j + 1 ]]    <- NULL
                next
        }

        treatmentcandidates$treatment1   <- 1

        #--- control ---#
        if( sum( subind1 ) == 0 ){
                subdata0                <- subdata
        }else{
                subdata0                <- subdata[ !( subdata$id %in% treatmentcandidates$id ), ]
        }
        subind0                  <- ( subdata0$start <= j & subdata0$stop > j )
        controlcandidates        <- subdata0[ subind0, ]
        if( nrow( controlcandidates ) == 0 ){
                matchinglist[[ j + 1 ]]    <- NULL
                print( j )
                next
        }
        controlcandidates$treatment1     <- 0

        rownames( treatmentcandidates ) <- rownames( controlcandidates ) <- NULL

        #--- matching ---#
        predata                  <- NULL
        predata                  <- rbind( predata, controlcandidates   )
        predata                  <- rbind( predata, treatmentcandidates )
        predata                  <- predata[ !is.na( predata$PS ), ]
        if( nrow( predata ) != 1 ){
                tryCatch( {
                        mout                    <- Match(
                                                        Y       = predata$outcome,
                                                        Tr      = predata$treatment1,
                                                        X       = predata$PS,
                                                        caliper = 0.2,
```

© 2021 Okubo M et al. *JAMA Network Open.*

```
                                                            ties    = F,
                                                            replace = F
                                                           )
                }, silent  = TRUE )
                if( sum( is.na( mout ) ) >= 1 ) {         # stopping rule
                        matchinglist[[ j + 1 ]]    <- NULL
                }else{
                        matchinglist[[ j + 1 ]]    <- try(
        # "matchinglist" is a list which includes observation number sets of matched pairs
                                                            data.frame(
                                                             cbind(
                                                              predata[ mout$index.treated, ]$obs,
                                                              predata[ mout$index.control, ]$obs
                                                             )
                                                            )
                                                           )
                        if( repstatus == "N" )exclude.ids               <- c( exclude.ids,
predata[ mout$index.treated, ]$id, predata[ mout$index.control, ]$id )
                }
        }else{
                matchinglist[[ j + 1 ]]    <- NULL

        }
        print( j )
}
matchinglistpreserve       <- matchinglist

names( matchinglist )      <- 1:( length( matchinglist ) )
for( h in ( 1:length( matchinglist ) )[ !sapply( matchinglist, is.null ) ] ){               # function to
add timing of match
  matchinglist[[ h ]]$X3   <- h - 1
}

matchedOBSset             <- ldply( matchinglist )                     # list to data.frame
matchedOBSset$pair        <- 1:nrow( matchedOBSset )                    # matchig pair indicator
colnames( matchedOBSset )  <- c( ".id","treatedobs", "controledobs", "timing" ,"pair" )
matchedOBSset.long        <- tidyr::gather( matchedOBSset, key = treatment1, value = obs, -
timing, -pair, -.id )   # convert to long format

matcheddata              <- merge(
                                    x     = matchedOBSset.long[ , - 1 ],
                                    y     = analysisdata[ , - which( names( analysisdata ) ==
"treatment1" ) ],
                                    by    = "obs",
                                    all.x = T
                                   )
```

© 2021 Okubo M et al. *JAMA Network Open.*

```
matcheddata$w             <- rep( rle( matcheddata$obs )$length, rle( matcheddata$obs )$length ) #
frequency weight

if(0){
        head( matcheddata )
}

#----------------------------------------------------------------------------------------------------#
# For the created matched data, the some statistical analysis methods such as
# GEE and conditional logistic methods will be applied by using standard R functions.
#----------------------------------------------------------------------------------------------------#
```

© 2021 Okubo M et al. *JAMA Network Open.*

**eFigure 1.** Patient Flow

ALS indicates advanced life support; DNR do-not resuscitate; EMS emergency medical services; OHCA out-of-hospital cardiac arrest.

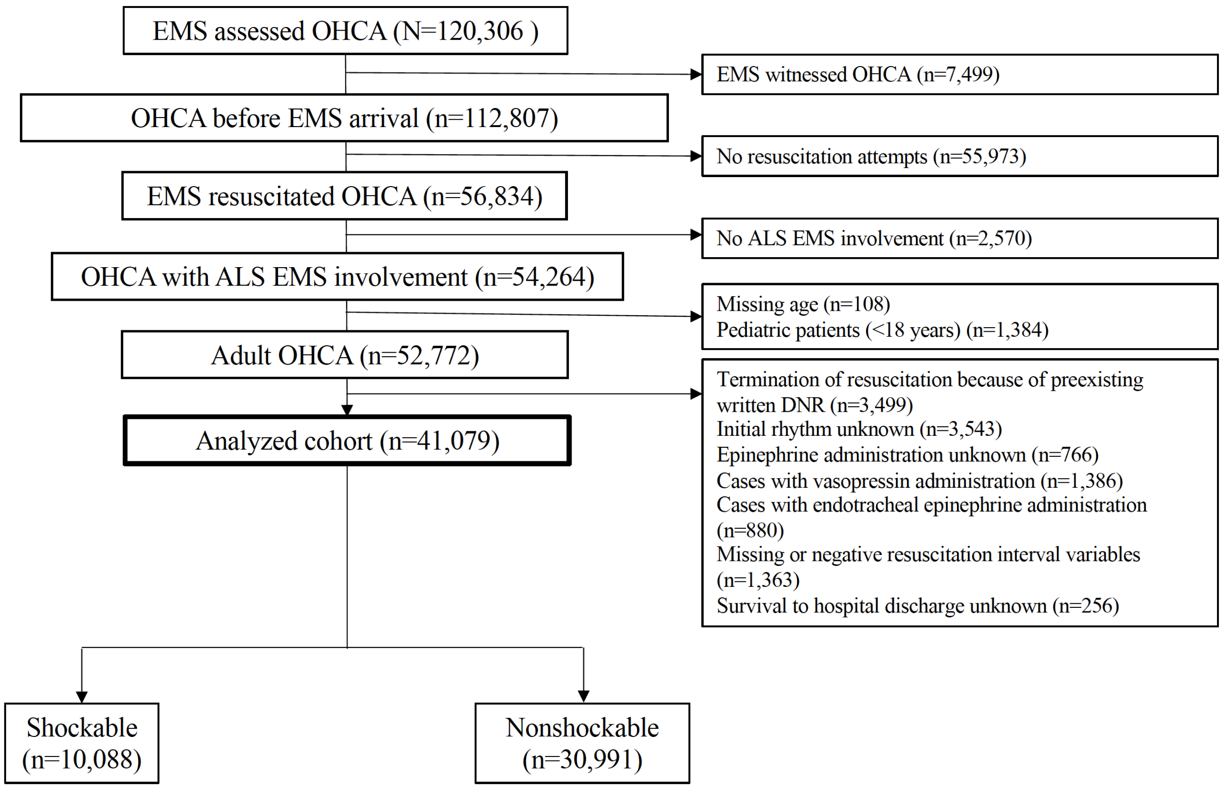


© 2021 Okubo M et al. *JAMA Network Open.*

**eFigure 2.** Association of epinephrine administration with survival to hospital discharge (A), favorable functional outcome at hospital discharge (B), and prehospital ROSC (C) by the timing of the administration for patients with shockable OHCA (matching without replacement).

Point estimates of the association of epinephrine with outcomes (solid lines) were reported with 95% CIs (dot lines), treating timing of epinephrine administration after ALS EMS as a continuous variable. Plots indicate point estimates of the association of epinephrine with outcomes with 95% CIs, treating timing as a categorical variable. The plots were placed at median time for each categorized time. We rounded decimal points of the numbers of imputed patients with favorable functional status (eFigure 2B).
eFigure 2A: Change of RR per minute = -4.6% (95% CI -7.7% to -1.4%, p-value for the interaction=0.005). eFigure 2B: Change of RR per minute = -4.8% (95% CI -8.4% to -1.0%, p-value for the interaction=0.02). eFigure 2C: Change of RR per minute = 0.7% (95% CI -1.0% to 2.5%, p-value for the interaction=0.409).
ALS indicates advanced life support; CI confidence interval; EMS emergency medical services; OHCA out-of-hospital cardiac arrest; ROSC return of spontaneous circulation.

**A**

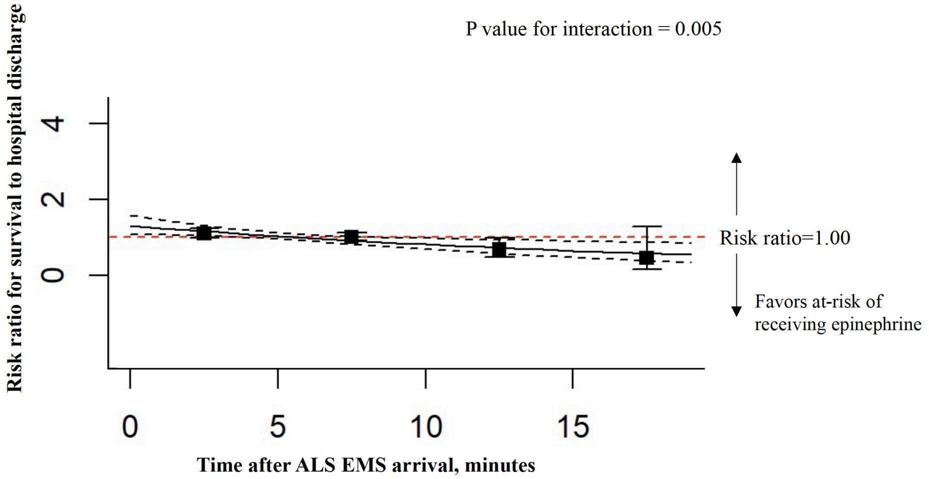


| Time after ALS EMS arrival, minutes | 0-5 | 5-10 | 10-15 | 15-20 |
|---|---|---|---|---|
| Epinephrine | 425/1494 (28.4%) | 540/2661 (20.3%) | 40/294 (13.6%) | 4/33 (12.1%) |
| At-risk of receiving epinephrine | 387/1494 (25.9%) | 538/2661 (20.2%) | 59/294 (20.1%) | 9/33 (27.3%) |
| Risk ratio (95% CI) | 1.10 (0.98-1.24) | 1.00 (0.9-1.11) | 0.68 (0.47-0.98) | 0.45 (0.16-1.28) |

© 2021 Okubo M et al. *JAMA Network Open.*

**B**

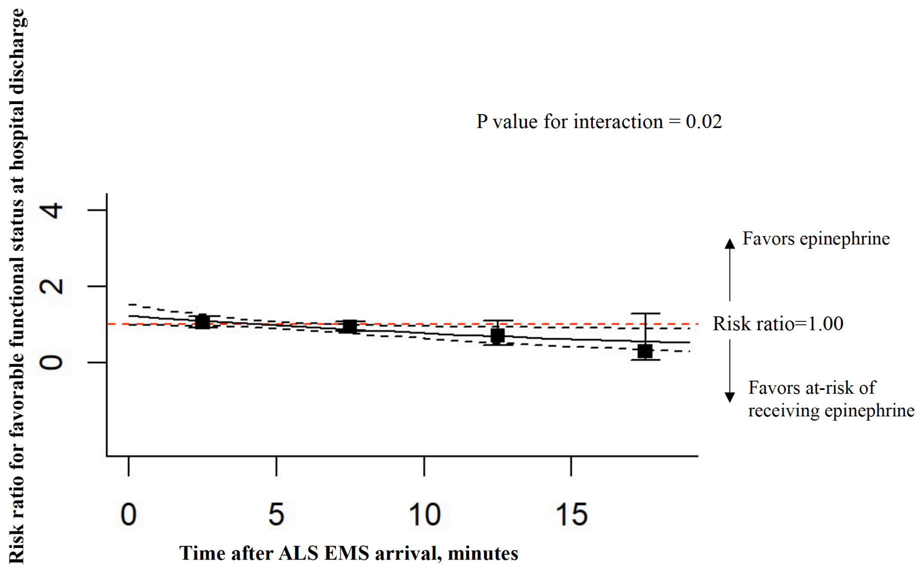


| **Time after ALS EMS arrival, minutes** | **0-5** | **5-10** | **10-15** | **15-20** |
|---|---|---|---|---|
| Epinephrine | 340/1494 (22.8%) | 412/2661 (15.5%) | 30/294 (10.2%) | 2/33 (6.1%) |
| At-risk of receiving epinephrine | 325/1494 (21.8%) | 438/2661 (16.5%) | 43/294 (14.6%) | 7/33 (21.2%) |
| Risk ratio (95% CI) | 1.05 (0.91-1.2) | 0.94 (0.83-1.07) | 0.70 (0.45-1.1) | 0.29 (0.07-1.28) |

© 2021 Okubo M et al. *JAMA Network Open.*

C

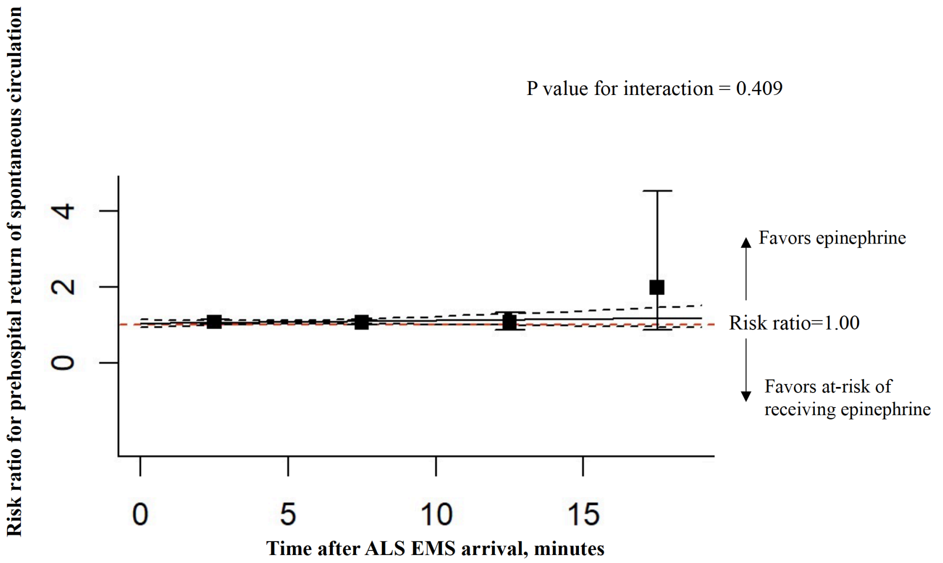


| **Time after ALS EMS arrival, minutes** | **0-5** | **5-10** | **10-15** | **15-20** |
|---|---|---|---|---|
| Epinephrine | 899/1494 (60.2%) | 1333/2661 (50.1%) | 108/294 (36.7%) | 12/33 (36.4%) |
| At-risk of receiving epinephrine | 832/1494 (55.7%) | 1255/2661 (47.2%) | 100/294 (34.0%) | 6/33 (18.2%) |
| Risk ratio (95% CI) | 1.08 (1.02-1.15) | 1.06 (1.01-1.12) | 1.08 (0.87-1.33) | 1.98 (0.87-4.53) |

© 2021 Okubo M et al. *JAMA Network Open.*

**eFigure 3.** Association of epinephrine administration with survival to hospital discharge (A), favorable functional outcome at hospital discharge (B), and prehospital ROSC (C) by the timing of the administration for patients with nonshockable OHCA (matching without replacement).

Point estimates of the association of epinephrine with outcomes (solid lines) were reported with 95% CIs (dot lines), treating timing of epinephrine administration after ALS EMS as a continuous variable. Plots indicate point estimates of the association of epinephrine with outcomes with 95% CIs, treating timing as a categorical variable. The plots were placed at median time for each categorized time. We rounded decimal points of the numbers of imputed patients with favorable functional status (eFigure 3B).
eFigure 3A: Change of RR per minute = -2.0% (95% CI -6.9% to 3.0%, p-value for the interaction=0.425). eFigure 3B: Change of RR per minute = -1.8% (95% CI -9.3% to 6.3%, p-value for the interaction=0.300). eFigure 3C: Change of RR per minute =0.03% (95% CI -1.3% to 1.4%, p-value for the interaction=0.963).
ALS indicates advanced life support; CI confidence interval; EMS emergency medical services; OHCA out-of-hospital cardiac arrest; ROSC return of spontaneous circulation.

**A**

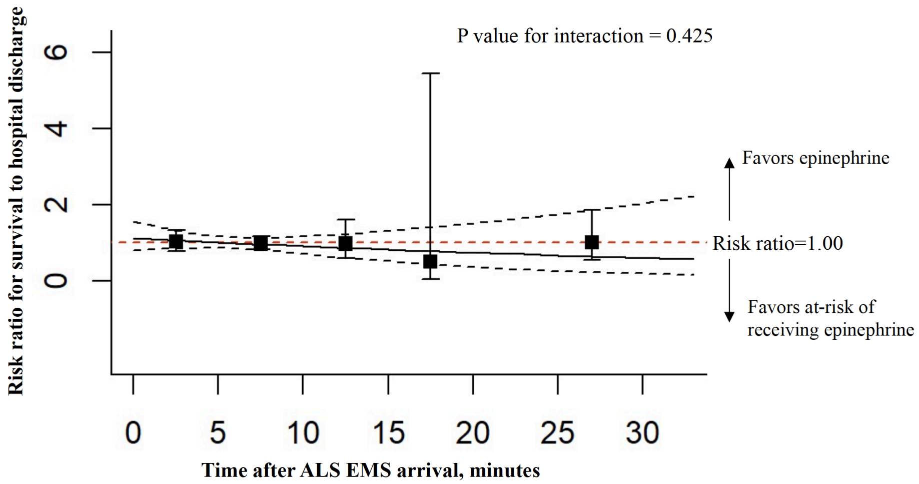


| **Time after ALS EMS arrival, minutes** | **0-5** | **5-10** | **10-15** | **15-20** | **> 20** |
|---|---|---|---|---|---|
| Epinephrine | 105/3681 (2.9%) | 230/9327 (2.5%) | 29/1594 (1.8%) | 1/131 (0.8%) | 0/20 (0%) |
| At-risk of receiving epinephrine | 103/3681 (2.8%) | 237/9327 (2.5%) | 30/1594 (1.9%) | 2/131 (1.5%) | 0/20 (0%) |
| Risk ratio (95% CI) | 1.02 (0.78-1.33) | 0.97 (0.81-1.16) | 0.97 (0.58-1.60) | 0.50 (0.05-5.44) | 1.00 (0.54-1.86) |

**B**

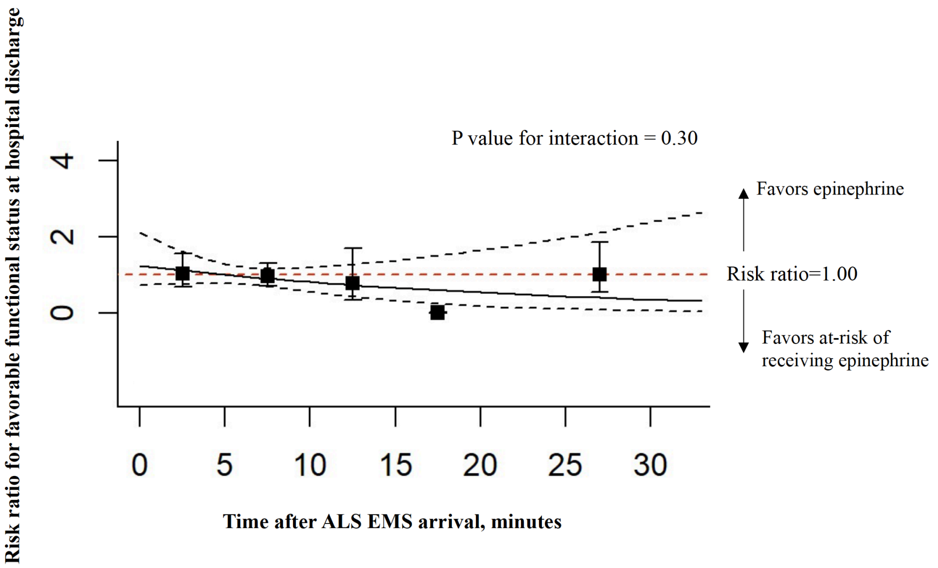


| Time after ALS EMS arrival, minutes | 0-5 | 5-10 | 10-15 | 15-20 | > 20 |
|---|---|---|---|---|---|
| Epinephrine | 51/3681 (1.4%) | 109/9327 (1.2%) | 13/1594 (0.8%) | 0/131 (0%) | 0/20 (0%) |
| At-risk of receiving epinephrine | 50/3681 (1.4%) | 114/9327 (1.2%) | 17/1594 (1.1%) | 1/131 (0.8%) | 0/20 (0%) |
| Risk ratio (95% CI) | 1.03 (0.68-1.56) | 0.96 (0.71-1.30) | 0.77 (0.34-1.70) | 0 (0-0.01) | 1.00 (0.54-1.86) |

© 2021 Okubo M et al. *JAMA Network Open.*

C

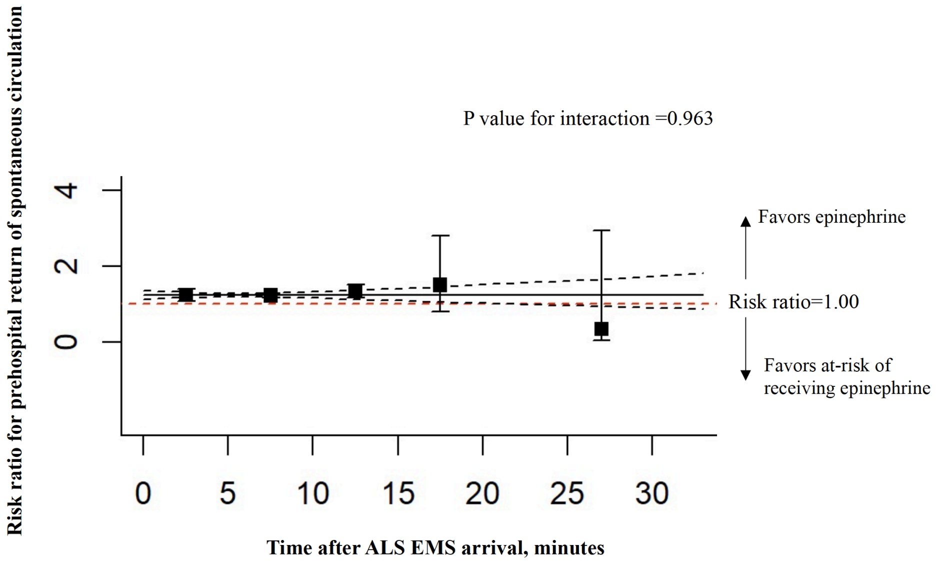


| **Time after ALS EMS arrival, minutes** | **0-5** | **5-10** | **10-15** | **15-20** | **> 20** |
|---|---|---|---|---|---|
| Epinephrine | 1244/3681 (33.8%) | 2888/9327 (31.0%) | 396/1594 (24.8%) | 21/131 (16.0%) | 1/20 (5.0%) |
| At-risk of receiving epinephrine | 1017/3681 (27.6%) | 2360/9327 (25.3%) | 298/1594 (18.7%) | 14/131 (10.7%) | 3/20 (15.0%) |
| Risk ratio (95% CI) | 1.23 (1.08-1.39) | 1.22 (1.17-1.28) | 1.33 (1.16-1.52) | 1.50 (0.80-2.80) | 0.33 (0.04-2.94) |

© 2021 Okubo M et al. *JAMA Network Open.*

**eFigure 4.** Association of epinephrine administration with survival to hospital discharge (A), favorable functional outcome at hospital discharge (B), and prehospital ROSC (C) by the timing of the administration for patients with shockable OHCA excluding those who had ROSC or TOR within 5 minutes after ALS EMS arrival on the scene.

Point estimates of the association of epinephrine with outcomes (solid lines) were reported with 95% CIs (dot lines), treating timing of epinephrine administration after ALS EMS as a continuous variable. Plots indicate point estimates of the association of epinephrine with outcomes with 95% CIs, treating timing as a categorical variable. The plots were placed at median time for each categorized time. We rounded decimal points of the numbers of imputed patients with favorable functional status (eFigure 4B).
eFigure 4A: Change of RR per minute = -6.3% (95% CI -8.5% to -4.1%, p-value for the interaction<0.001). eFigure 4B: Change of RR per minute = -7.4% (95% CI -10.1% to -4.7%, p-value for the interaction<0.001). eFigure 4C: Change of RR per minute = 0.4% (95% CI -0.9 to 1.7%, p-value for the interaction=0.52).
ALS indicates advanced life support; CI confidence interval; EMS emergency medical services; OHCA out-of-hospital cardiac arrest; ROSC return of spontaneous circulation; TOR termination of resuscitation.

**A**

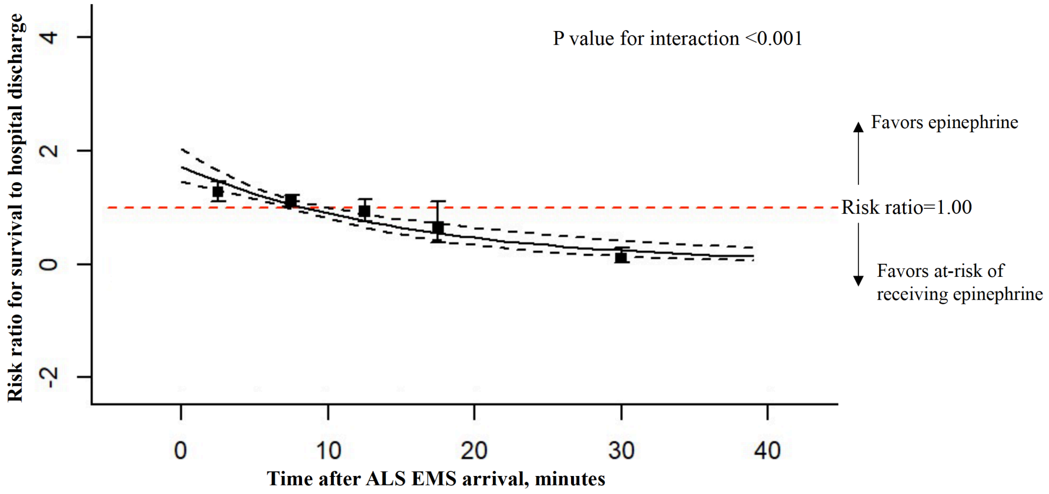


| Time after ALS EMS arrival, minutes | 0-5 | 5-10 | 10-15 | 15-20 | > 20 |
|---|---|---|---|---|---|
| Epinephrine | 420/1572 (26.7%) | 877/4540 (19.3%) | 185/1546 (12.0%) | 27/369 (7.3%) | 5/118 (4.2%) |
| At-risk of receiving epinephrine | 370/1572 (23.5%) | 898/4540 (19.8%) | 226/1546 (14.6%) | 53/369 (14.4%) | 25/118 (21.2%) |
| Risk ratio (95% CI) | 1.27 (1.12-1.46) | 1.11 (1.01-1.22) | 0.94 (0.76-1.15) | 0.66 (0.39-1.11) | 0.10 (0.04-0.29) |

© 2021 Okubo M et al. *JAMA Network Open.*

**B**

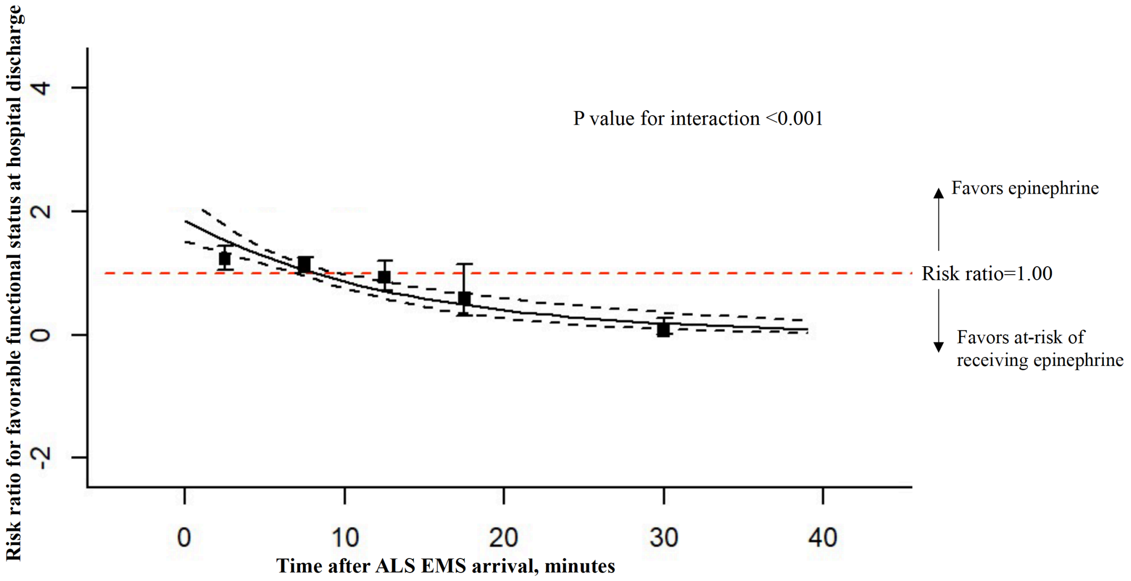


| **Time after ALS EMS arrival, minutes** | **0-5** | **5-10** | **10-15** | **15-20** | **> 20** |
|---|---|---|---|---|---|
| Epinephrine | 337/1572 (21.4%) | 687/4540 (15.1%) | 132/1546 (8.5%) | 18/369 (4.9%) | 3/118 (2.5%) |
| At-risk of receiving epinephrine | 307/1572 (19.5%) | 708/4540 (15.6%) | 163/1546 (10.5%) | 40/369 (10.8%) | 24/118 (20.3%) |
| Risk ratio (95% CI) | 1.23 (1.06-1.44) | 1.13 (1.01-1.26) | 0.93 (0.72-1.20) | 0.60 (0.31-1.15) | 0.06 (0.01-0.26) |

© 2021 Okubo M et al. *JAMA Network Open.*

**C**

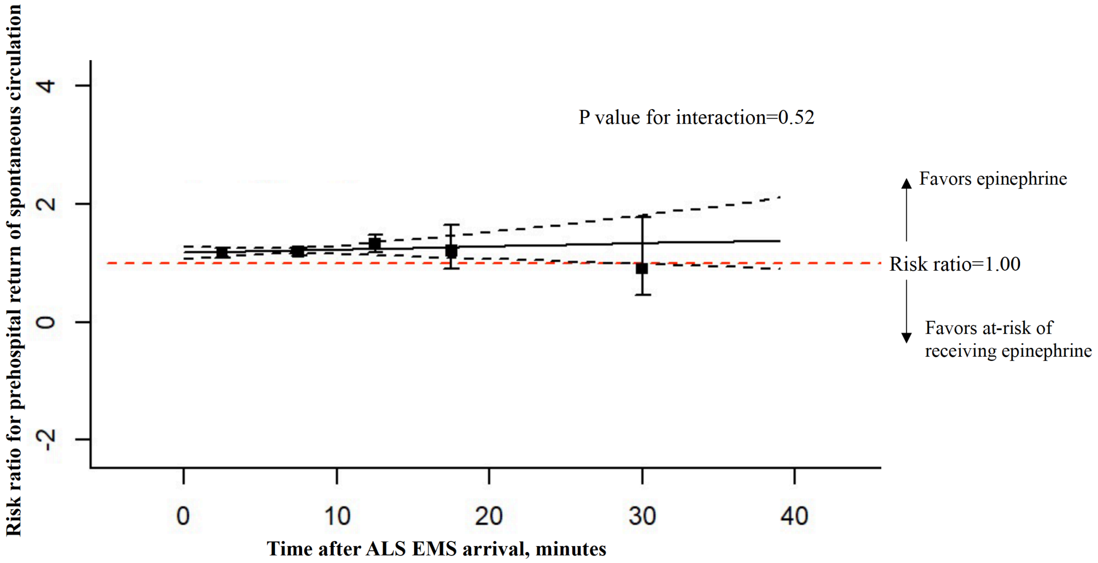


| **Time after ALS EMS arrival, minutes** | **0-5** | **5-10** | **10-15** | **15-20** | **> 20** |
|---|---|---|---|---|---|
| Epinephrine | 922/1572 (58.7%) | 2289/4540 (50.4%) | 579/1546 (37.5%) | 95/369 (25.7%) | 19/118 (16.1%) |
| At-risk of receiving epinephrine | 832/1572 (52.9%) | 2083/4540 (45.9%) | 473/1546 (30.6%) | 75/369 (20.3%) | 16/118 (13.6%) |
| Risk ratio (95% CI) | 1.18 (1.10-1.26) | 1.19 (1.13-1.25) | 1.32 (1.18-1.48) | 1.21 (0.90-1.64) | 0.90 (0.46-1.78) |

© 2021 Okubo M et al. *JAMA Network Open.*

**eFigure 5.** Association of epinephrine administration with survival to hospital discharge (A), favorable functional outcome at hospital discharge (B), and prehospital ROSC (C) by the timing of the administration for patients with nonshockable OHCA excluding those who had ROSC or TOR within 5 minutes after ALS EMS arrival on the scene.

Point estimates of the association of epinephrine with outcomes (solid lines) were reported with 95% CIs (dot lines), treating timing of epinephrine administration after ALS EMS as a continuous variable. Plots indicate point estimates of the association of epinephrine with outcomes with 95% CIs, treating timing as a categorical variable. The plots were placed at median time for each categorized time. We rounded decimal points of the numbers of imputed patients with favorable functional status (eFigure 5B).
eFigure 5A: Change of RR per minute = -3.2% (95% CI -6.3% to -0.03%, p-value for the interaction=0.048). eFigure 5B: Change of RR per minute = -5.0% (95% CI -9.8% to -0.07%, p-value for the interaction=0.03). eFigure 5C: Change of RR per minute = 1.3% (95% CI 0.4% to 2.3%, p-value for the interaction=0.007).
ALS indicates advanced life support; CI confidence interval; EMS emergency medical services; OHCA out-of-hospital cardiac arrest; ROSC return of spontaneous circulation; TOR termination of resuscitation.

**A**

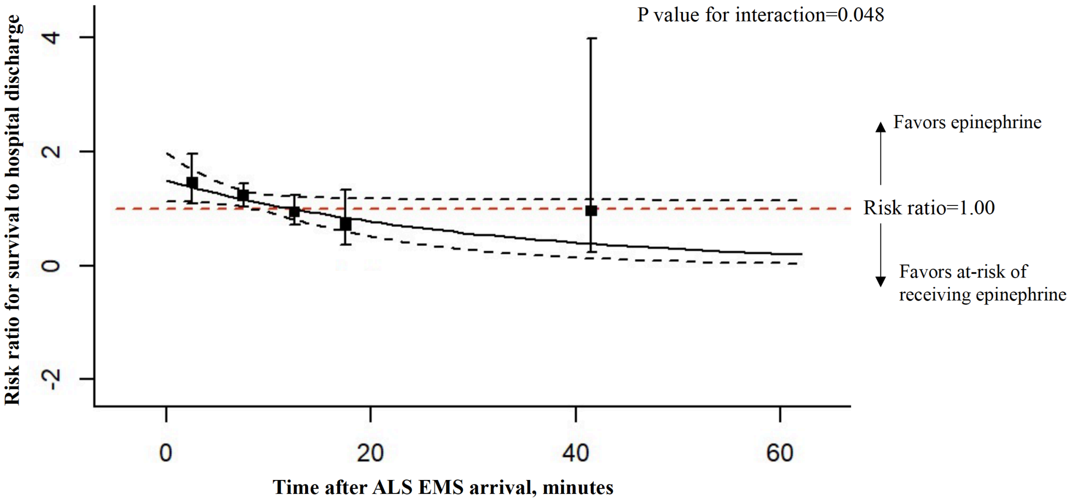


| **Time after ALS EMS arrival, minutes** | **0-5** | **5-10** | **10-15** | **15-20** | **> 20** |
|---|---|---|---|---|---|
| Epinephrine | 100/3822 (2.6%) | 361/14756 (2.4%) | 117/6650 (1.8%) | 21/1872 (1.1%) | 4/727 (0.6%) |
| At-risk of receiving epinephrine | 93/3822 (2.4%) | 341/14756 (2.3%) | 134/6650 (2%) | 29/1872 (1.5%) | 7/727 (1.0%) |
| Risk ratio (95% CI) | 1.46 (1.09-1.97) | 1.23 (1.04-1.44) | 0.94 (0.72-1.23) | 0.70 (0.37-1.33) | 0.96 (0.23-3.98) |

© 2021 Okubo M et al. *JAMA Network Open.*

**B**

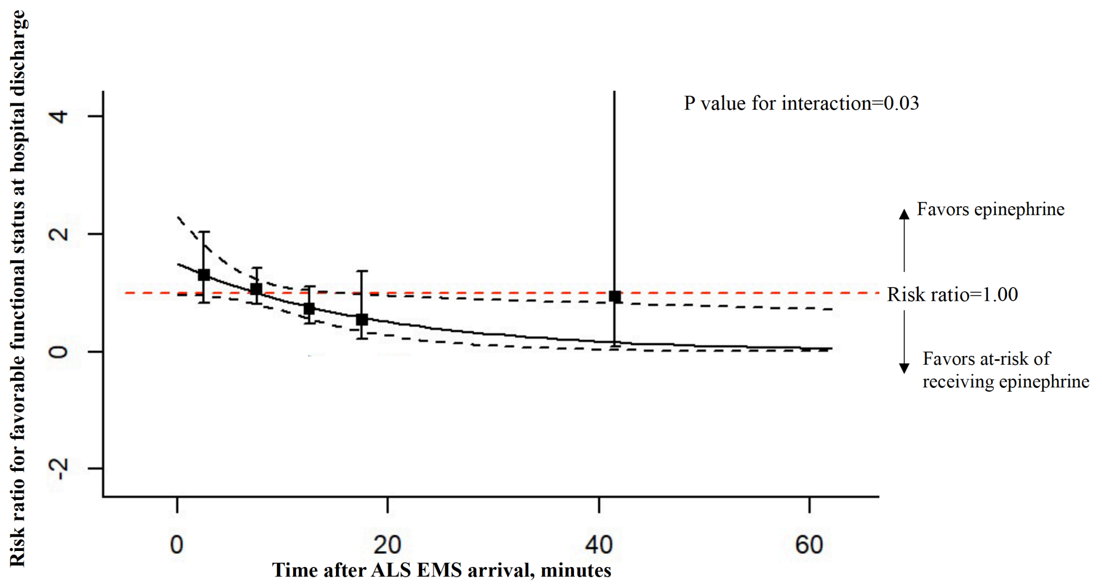


| Time after ALS EMS arrival, minutes | 0-5 | 5-10 | 10-15 | 15-20 | > 20 |
|---|---|---|---|---|---|
| Epinephrine | 49/3822 (1.3%) | 150/14756 (1.0%) | 49/6650 (0.7%) | 9/1872 (0.5%) | 1/727 (0.1%) |
| At-risk of receiving epinephrine | 52/3822 (1.4%) | 164/14756 (1.1%) | 75/6650 (1.1%) | 16/1872 (0.9%) | 3/727 (0.4%) |
| Risk ratio (95% CI) | 1.30 (0.83-2.03) | 1.06 (0.80-1.41) | 0.73 (0.48-1.11) | 0.54 (0.21-1.36) | 0.94 (0.09-9.69) |

© 2021 Okubo M et al. *JAMA Network Open.*

**C**

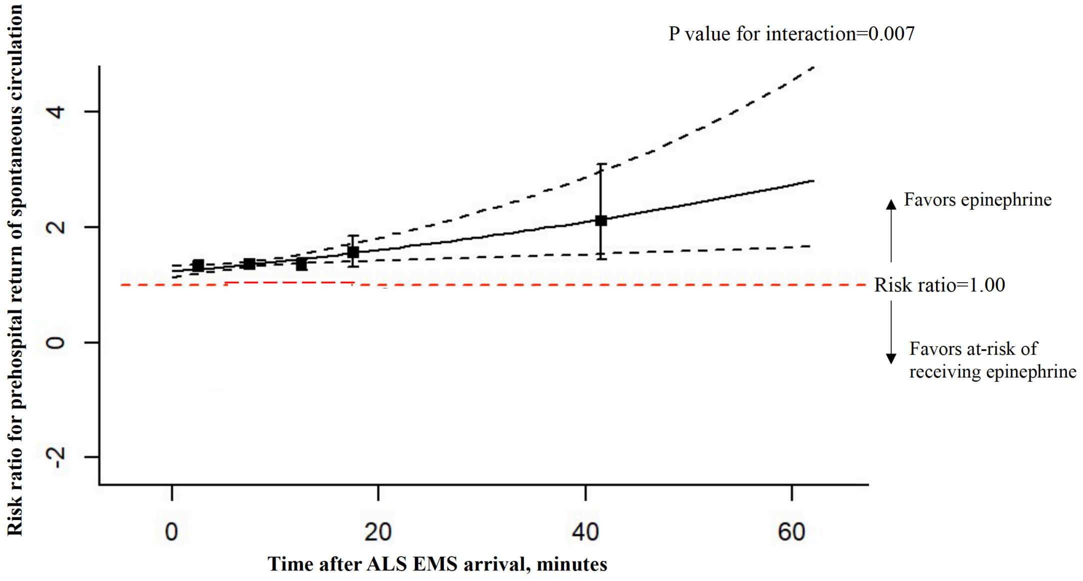


| **Time after ALS EMS arrival, minutes** | **0-5** | **5-10** | **10-15** | **15-20** | **> 20** |
|---|---|---|---|---|---|
| Epinephrine | 1261/3822 (33.0%) | 4531/14756 (30.7%) | 1647/6650 (24.8%) | 348/1872 (18.6%) | 93/727 (12.8%) |
| At-risk of receiving epinephrine | 1038/3822 (27.2%) | 3520/14756 (23.9%) | 1216/6650 (18.3%) | 218/1872 (11.6%) | 42/727 (5.8%) |
| Risk ratio (95% CI) | 1.33 (1.23-1.43) | 1.36 (1.31-1.42) | 1.36 (1.26-1.46) | 1.56 (1.31-1.85) | 2.11 (1.44-3.1) |

© 2021 Okubo M et al. *JAMA Network Open.*

**eFigure 6.** Association of epinephrine administration with survival to hospital discharge (A), favorable functional outcome at hospital discharge (B), and prehospital ROSC (C) by the timing of the administration for patients with bystander witnessed shockable OHCA.

Point estimates of the association of epinephrine with outcomes (solid lines) were reported with 95% CIs (dot lines), treating timing of epinephrine administration after ALS EMS as a continuous variable. Plots indicate point estimates of the association of epinephrine with outcomes with 95% CIs, treating timing as a categorical variable. The plots were placed at median time for each categorized time. We rounded decimal points of the numbers of imputed patients with favorable functional status (eFigure 6B).
eFigure 6A: Change of RR per minute = -5.4% (95% CI -7.8% to -3.0%, p-value for the interaction<0.001). eFigure 6B: Change of RR per minute = -6.5% (95% CI -9.3% to -3.5%, p-value for the interaction<0.001). eFigure 6C: Change of RR per minute = 0.4% (95% CI -1.1% to 2.0%%, p-value for the interaction=0.56).
ALS indicates advanced life support; CI confidence interval; EMS emergency medical services; OHCA out-of-hospital cardiac arrest; ROSC return of spontaneous circulation.

**A**

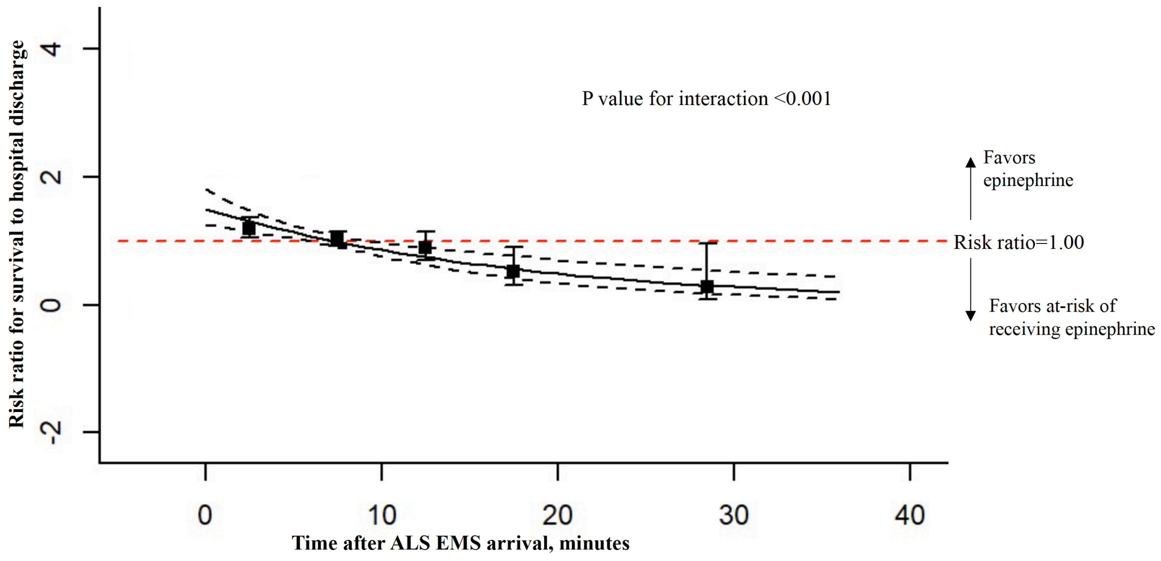


| Time after ALS EMS arrival, minutes | 0-5 | 5-10 | 10-15 | 15-20 | > 20 |
|---|---|---|---|---|---|
| Epinephrine | 364/1113 (32.7%) | 656/2967 (22.1%) | 133/945 (14.1%) | 21/233 (9.0%) | 4/68 (5.9%) |
| At-risk of receiving epinephrine | 349/1113 (31.4%) | 718/2967 (24.2%) | 157/945 (16.6%) | 47/233 (20.2%) | 16/68 (23.5%) |
| Risk ratio (95% CI) | 1.19 (1.04-1.37) | 1.03 (0.92-1.14) | 0.90 (0.70-1.14) | 0.52 (0.30-0.91) | 0.27 (0.08-0.95) |

© 2021 Okubo M et al. *JAMA Network Open.*

**B**

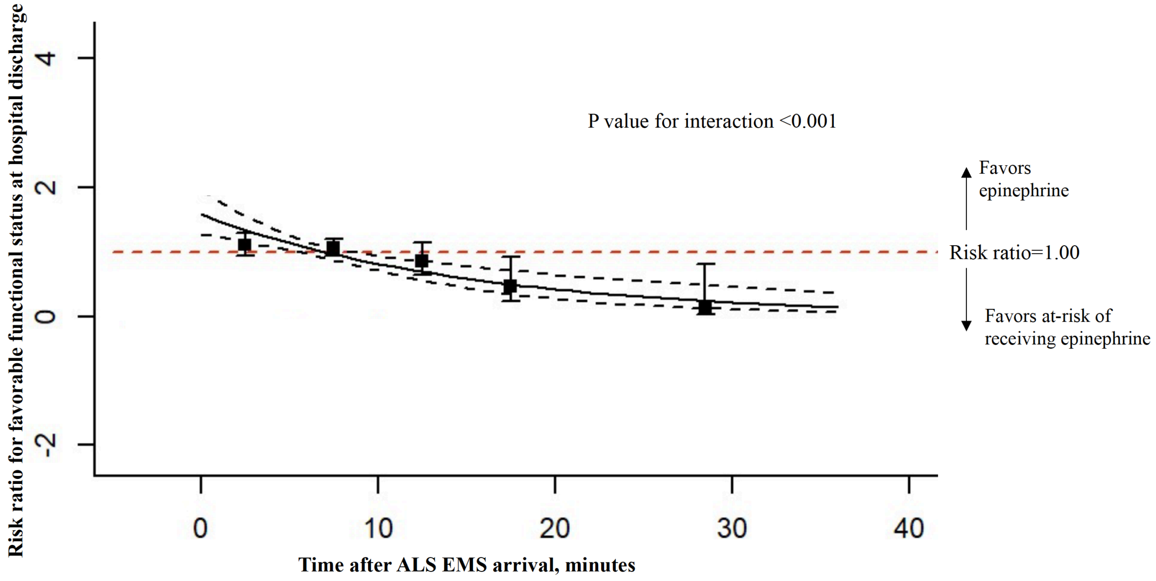


| **Time after ALS EMS arrival, minutes** | **0-5** | **5-10** | **10-15** | **15-20** | **> 20** |
|---|---|---|---|---|---|
| Epinephrine | 296/1113 (26.6%) | 521/2967 (17.6%) | 101/945 (10.7%) | 14/233 (6.0%) | 2/68 (3.0%) |
| At-risk of receiving epinephrine | 309/1113 (27.8%) | 578/2967 (19.5%) | 121/945 (12.8%) | 37/233 (15.9%) | 15/68 (22.1%) |
| Risk ratio (95% CI) | 1.10 (0.95-1.29) | 1.06 (0.93-1.19) | 0.86 (0.64-1.15) | 0.46 (0.23-0.91) | 0.14 (0.03-0.80) |

© 2021 Okubo M et al. *JAMA Network Open.*

**C**

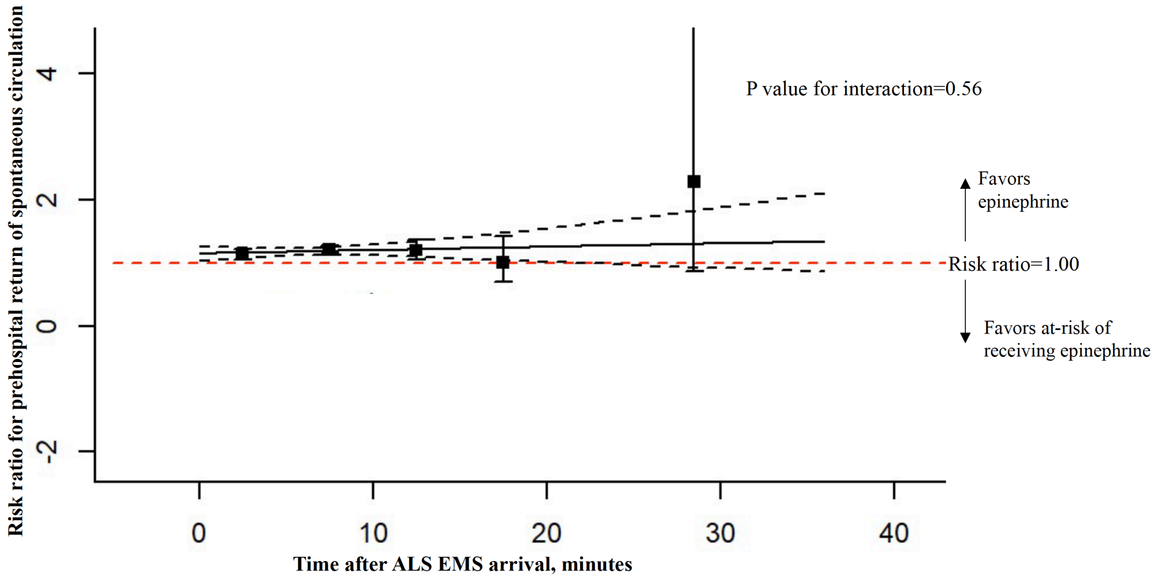


| Time after ALS EMS arrival, minutes | 0-5 | 5-10 | 10-15 | 15-20 | > 20 |
|---|---|---|---|---|---|
| Epinephrine | 719/1113 (64.6%) | 1578/2967 (53.2%) | 385/945 (40.7%) | 63/233 (27.0%) | 16/68 (23.5%) |
| At-risk of receiving epinephrine | 677/1113 (60.8%) | 1455/2967 (49.0%) | 330/945 (34.9%) | 62/233 (26.6) | 7/68 (10.3%) |
| Risk ratio (95% CI) | 1.13 (1.05-1.22) | 1.20 (1.14-1.28) | 1.20 (1.05-1.37) | 1.00 (0.70-1.42) | 2.29 (0.87-6.05) |

© 2021 Okubo M et al. *JAMA Network Open.*

**eFigure 7.** Association of epinephrine administration with survival to hospital discharge (A), favorable functional outcome at hospital discharge (B), and prehospital ROSC (C) for patients with bystander witnessed nonshockable OHCA.

Point estimates of the association of epinephrine with outcomes (solid lines) were reported with 95% CIs (dot lines), treating timing of epinephrine administration after ALS EMS as a continuous variable. Plots indicate point estimates of the association of epinephrine with outcomes with 95% CIs, treating timing as a categorical variable. The plots were placed at median time for each categorized time. We rounded decimal points of the numbers of imputed patients with favorable functional status (eFigure 7B).
eFigure 7A: Change of RR per minute = -2.8% (95% CI -7.2% to 1.8%, p-value for the interaction=0.23). eFigure 7B: Change of RR per minute = -5.8% (95% CI -11.7% to 0.4%, p-value for the interaction=0.09). eFigure 7C: Change of RR per minute = 1.6% (95% CI 0.4% to 2.9%, p-value for the interaction=0.01)
ALS indicates advanced life support; CI confidence interval; EMS emergency medical services; OHCA out-of-hospital cardiac arrest; ROSC return of spontaneous circulation.

**A**

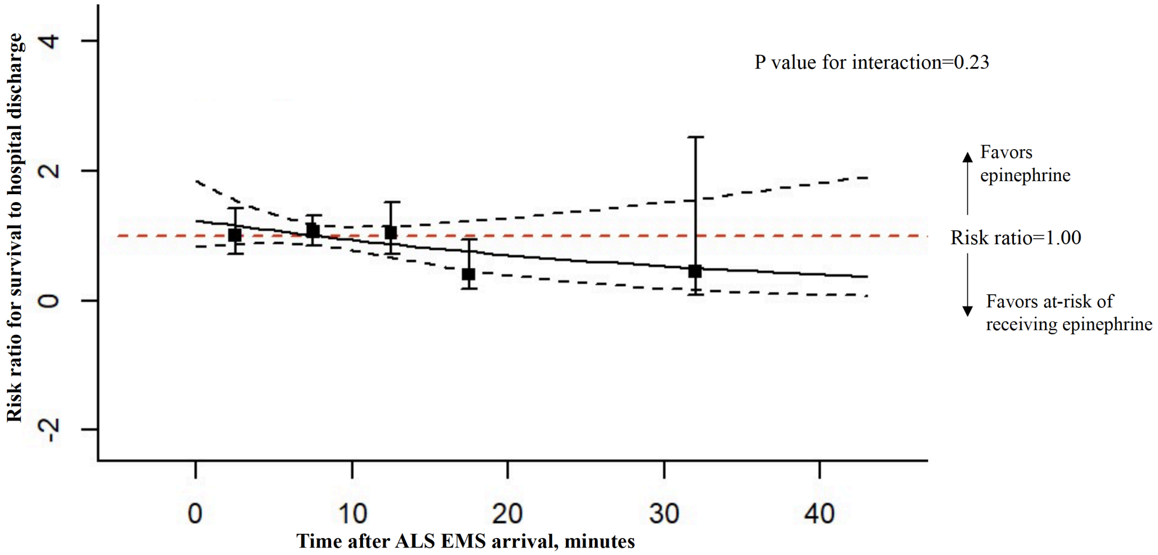


| **Time after ALS EMS arrival, minutes** | **0-5** | **5-10** | **10-15** | **15-20** | **> 20** |
|---|---|---|---|---|---|
| Epinephrine | 63/1366 (4.6%) | 183/4955 (3.7%) | 65/2250 (2.9%) | 10/607 (1.6%) | 2/240 (0.8%) |
| At-risk of receiving epinephrine | 75/1366 (5.5%) | 209/4955 (4.2%) | 70/2250 (3.1%) | 19/607 (3.1%) | 7/240 (2.9%) |
| Risk ratio (95% CI) | 1.00 (0.71-1.42) | 1.06 (0.85-1.31) | 1.05 (0.72-1.51) | 0.40 (0.17-0.95) | 0.44 (0.08-2.51) |

© 2021 Okubo M et al. *JAMA Network Open.*

**B**

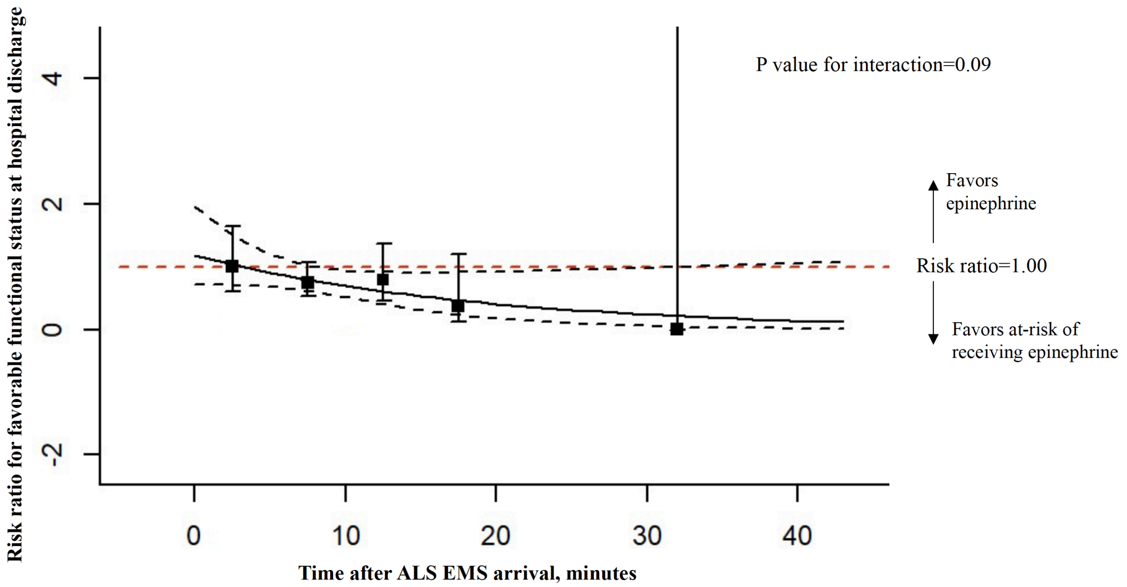


| Time after ALS EMS arrival, minutes | 0-5 | 5-10 | 10-15 | 15-20 | > 20 |
|---|---|---|---|---|---|
| Epinephrine | 34/1366 (2.5%) | 76/4955 (1.5%) | 33/2250 (1.5%) | 5/607 (0.8%) | 0/240 (0%) |
| At-risk of receiving epinephrine | 42/1366 (3.1%) | 122/4955 (2.5%) | 43/2250 (1.9%) | 14/607 (2.3%) | 3/240 (1.3%) |
| Risk ratio (95% CI) | 1.00 (0.60-1.65) | 0.75 (0.52-1.07) | 0.79 (0.46-1.36) | 0.37 (0.12-1.19 | 0 (0-34816756) |

© 2021 Okubo M et al. *JAMA Network Open.*

C

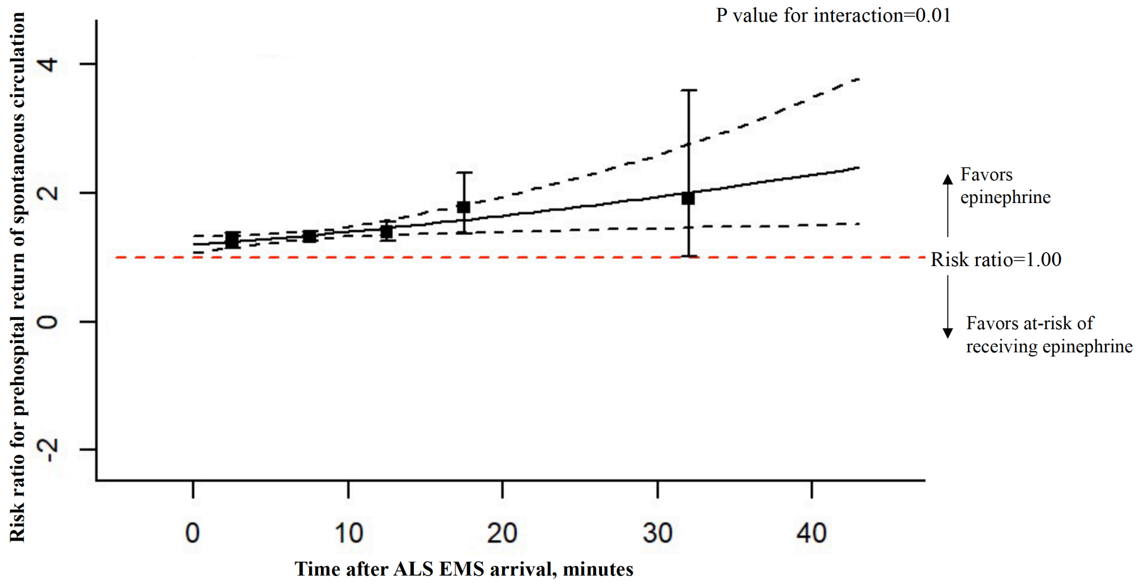


| **Time after ALS EMS arrival, minutes** | **0-5** | **5-10** | **10-15** | **15-20** | **> 20** |
|---|---|---|---|---|---|
| Epinephrine | 636/1366 (46.6%) | 2137/4955 (43.1%) | 751/2250 (33.4%) | 151/607 (24.9%) | 33/240 (13.8%) |
| At-risk of receiving epinephrine | 546/1366 (40%) | 1706/4955 (34.4%) | 562/2250 (25.0%) | 86/607 (14.2%) | 16/240 (6.7%) |
| Risk ratio (95% CI) | 1.26 (1.15-1.39) | 1.32 (1.25-1.40) | 1.39 (1.26-1.55) | 1.78 (1.36-2.32) | 1.91 (1.02-3.60) |

© 2021 Okubo M et al. *JAMA Network Open.*

**eTable 1**. Characteristics of adult patients with out-of-hospital cardiac arrest with epinephrine and at risk of receiving epinephrine in time-dependent propensity score matched cohort (matching without replacement)

| | Shockable rhythms | | | Nonshockable rhythms | | |
|---|---|---|---|---|---|---|
| | At-risk of receiving epinephrine (n=4482) | Epinephrine (n=4482) | standardized difference | At-risk of receiving epinephrine (n=14753) | Epinephrine (n=14753) | standardized difference |
| Age, median (IQR), y | 65 (55-76) | 64 (54-75) | 0.053 | 68 (55-80) | 68 (55-81) | 0.01 |
| Sex | | | 0.068 | | | 0.021 |
| Male | 3413 (76.1) | 3530 (78.8) | | 9021 (61.1) | 8981 (60.9) | |
| Unknown | 4 (0.1) | 1 (0.0) | | 3 (0.0) | 9 (0.1) | |
| Race | | | 0.032 | | | 0.004 |
| White | 1105 (24.7) | 1167 (26.0) | | 3541 (24.0) | 3567 (24.2) | |
| Non-white | 3377 (75.3) | 3315 (74.0) | | 11212 (76.0) | 11186 (75.8) | |
| Etiology | | | 0.025 | | | 0.025 |
| Cardiac | 4392 (98.0) | 4407 (98.3) | | 13671 (92.7) | 13573 (92.0) | |
| Non-cardiac | 89 (2.0) | 74 (1.7) | | 1082 (7.3) | 1180 (8.0) | |
| Unknown | 1 (0.0) | 1 (0.0) | | | | |
| Initial rhythm | | | | | | 0.055 |
| PEA | N/A | N/A | | 4216 (28.6) | 4585 (31.1) | |
| Asystole | N/A | N/A | | 10537 (71.4) | 10168 (68.9) | |
| Location | | | 0.117 | | | 0.055 |
| Street/highway | 405 (9.0) | 426 (9.5) | | 420 (2.8) | 433 (2.9) | |
| Public building | 91 (2.0) | 97 (2.2) | | 97 (0.7) | 93 (0.6) | |
| Place of recreation | 171 (3.8) | 205 (4.6) | | 193 (1.3) | 161 (1.1) | |
| Industrial place | 97 (2.2) | 100 (2.2) | | 69 (0.5) | 65 (0.4) | |
| Home | 2910 (64.9) | 2824 (63.0) | | 11129 (75.4) | 11186 (75.8) | |
| Farm/ranch | 7 (0.2) | 0 (0.0) | | 16 (0.1) | 12 (0.1) | |
| Healthcare facility | 73 (1.6) | 74 (1.7) | | 276 (1.9) | 329 (2.2) | |

© 2021 Okubo M et al. *JAMA Network Open.*

| | | | | | | |
|---|---|---|---|---|---|---|
| Residential institution | 195 (4.4) | 136 (3.0) | | 1891 (12.8) | 1730 (11.7) | |
| Other public property | 499 (11.1) | 575 (12.8) | | 587 (4.0) | 676 (4.6) | |
| Other non-public property | 22 (0.5) | 34 (0.8) | | 47 (0.3) | 40 (0.3) | |
| Unknown | 12 (0.3) | 11 (0.2) | | 28 (0.2) | 28 (0.2) | |
| Witnessed collapse | | | 0.058 | | | 0.021 |
| Bystander | 2903 (64.8) | 2999 (66.9) | | 4916 (33.3) | 5060 (34.3) | |
| None | 1486 (33.2) | 1373 (30.6) | | 9429 (63.9) | 9288 (63.0) | |
| Unknown | 93 (2.1) | 110 (2.5) | | 408 (2.8) | 405 (2.7) | |
| Layperson CPR | | | | | | 0.033 |
| Yes | 2460 (54.9) | 2705 (60.4) | | 6806 (46.1) | 7030 (47.7) | |
| No | 1932 (43.1) | 1707 (38.1) | | 7640 (51.8) | 7399 (50.2) | |
| Unknown | 90 (2.0) | 70 (1.6) | | 307 (2.1) | 324 (2.2) | |
| Shock delivery before ALS arrival | | | 0.119 | | | 0.023 |
| Yes | 1178 (26.3) | 1419 (31.7) | | 151 (1.0) | 165 (1.1) | |
| No | 3304 (73.7) | 3063 (68.3) | | 14602 (99.0) | 14588 (98.9) | |
| EMS response time (interval between 9-1-1 call and first EMS arrival), median (IQR), minutes | 5.5 (4.3-7.0) | 5.5 (4.2-7.0) | 0.037 | 5.4 (4.2-7.0) | 5.4 (4.2-7.0) | 0.016 |
| Shock delivery after ALS arrival | | | | | | |
| Yes | 3263 (72.8) | 2944 (65.7) | 0.155 | 2245 (15.2) | 2337 (15.8) | 0.017 |
| Interval between ALS arrival and shock delivery, median (IQR), minutes | 4.1 (2.7-5.9) | 3.8 (2.4-5.7) | 0.075 | 14.6 (8.7-21.0) | 12.5 (8.2-18.5) | 0.171 |
| Advanced airway management | | | | | | |
| Yes | 3445 (76.9) | 3580 (79.9) | 0.073 | 10933 (74.1) | 11473 (77.8) | 0.086 |
| Interval between ALS arrival and AAM, median (IQR), minutes | 11.0 (7.1-16.0) | 10.0 (6.7-14.0) | 0.189 | 11.2 (8.0-13.0) | 10.0 (7.0-13.6) | 0.211 |
| Departure from the scene | | | | | | |
| Yes | 3606 (80.5) | 3546 (79.1) | 0.033 | 7457 (50.5) | 7081 (48.0) | 0.051 |

© 2021 Okubo M et al. *JAMA Network Open.*

| | | | | | | |
|---|---|---|---|---|---|---|
| Interval between ALS arrival and departure from the scene, median (IQR), minutes | 24.8 (19.0-32.0) | 24.0 (18.9-30.4) | 0.097 | 25.5 (19.2-33.1) | 24.9 (19.1-31.5) | 0.09 |

IQR indicates interquartile range; PEA, pulseless electrical activity; CPR, cardiopulmonary resuscitation; ALS, advanced life support; EMS, emergency medical services; and AAM, advanced airway management

© 2021 Okubo M et al. *JAMA Network Open.*

**eTable 2**. Outcomes in time-dependent propensity score matched cohort (matching without replacement)

| Outcomes | No (%) patients with outcome/total patients | | Risk ratio (95% CI) |
|---|---|---|---|
| | At-risk of receiving epinephrine | Epinephrine | |
| Shockable rhythms | | | |
| Survival to hospital discharge | 993/4482 (22.2%) | 1009/4482 (22.5%) | 1.02 (0.94-1.10) |
| Favorable functional outcome at hospital discharge | 813/4482 (18.1%) | 784/4482 (17.5%) | 0.96 (0.88-1.06) |
| Prehospital ROSC | 2193/4482 (48.9%) | 2352/4482 (52.5%) | 1.07 (1.03-1.12) |
| Nonshockable rhythms | | | |
| Survival to hospital discharge | 372/14753 (2.5%) | 365/14753 (2.5%) | 0.98 (0.85-1.13) |
| Favorable functional outcome at hospital discharge | 182/14753 (1.2%) | 173/14753 (1.2%) | 0.95 (0.76-1.20) |
| Prehospital ROSC | 3692/14753 (25.0%) | 4550/14753 (30.8%) | 1.23 (1.19-1.28) |

ROSC indicates return of spontaneous circulation.

© 2021 Okubo M et al. *JAMA Network Open.*

**eTable 3.** Characteristics of adult patients with out-of-hospital cardiac arrest with and without epinephrine in original cohort, excluding those who had ROSC or TOR within 5 minutes after ALS EMS arrival

| | Shockable rhythms | | | Nonshockable rhythms | | |
|---|---|---|---|---|---|---|
| | No epinephrine (n=1051) | Epinephrine (n=8158) | standardized difference | No epinephrine (n=2223) | Epinephrine (n=27841) | standardized difference |
| Age, median (IQR), y | 63 (53-72) | 65 (55-76) | 0.164 | 70 (56-82) | 68 (55-80) | 0.08 |
| Sex | | | 0.167 | | | 0.149 |
| Male | 744 (70.8) | 6368 (78.1) | | 1200 (54.0) | 17071 (61.3) | |
| Unknown | 1 (0.1) | 4 (0.0) | | 2 (0.1) | 12 (0.0) | |
| Race | | | 0.006 | | | 0.174 |
| White | 264 (25.1) | 2071 (25.4) | | 388 (17.5) | 6830 (24.5) | |
| Non-white | 787 (74.9) | 6087 (74.6) | | 1835 (82.5) | 21011 (75.5) | |
| Etiology | | | 0.051 | | | 0.151 |
| Cardiac | 1026 (97.6) | 8013 (98.2) | | 1960 (88.2) | 25766 (92.5) | |
| Non-cardiac | 24 (2.3) | 144 (1.8) | | 262 (11.8) | 2075 (7.5) | |
| Unknown | 1 (0.1) | 1 (0.0) | | 1 (0.0) | 0 (0.0) | |
| Initial rhythm | | | | | | 0.155 |
| PEA | N/A | N/A | | 819 (36.8) | 8226 (29.5) | |
| Asystole | N/A | N/A | | 1404 (63.2) | 19615 (70.5) | |
| Location | | | 0.25 | | | 0.119 |
| Street/highway | 120 (11.4) | 738 (9.0) | | 86 (3.9) | 790 (2.8) | |
| Public building | 39 (3.7) | 168 (2.1) | | 18 (0.8) | 177 (0.6) | |
| Place of recreation | 63 (6.0) | 339 (4.2) | | 37 (1.7) | 324 (1.2) | |
| Industrial place | 20 (1.9) | 179 (2.2) | | 2 (0.1) | 131 (0.5) | |
| Home | 564 (53.7) | 5282 (64.7) | | 1644 (74.0) | 21074 (75.7) | |
| Farm/ranch | 0 (0.0) | 7 (0.1) | | 2 (0.1) | 26 (0.1) | |
| Healthcare facility | 24 (2.3) | 131 (1.6) | | 57 (2.6) | 564 (2.0) | |
| Residential institution | 46 (4.4) | 300 (3.7) | | 276 (12.4) | 3429 (12.3) | |
| Other public property | 159 (15.1) | 946 (11.6) | | 89 (4.0) | 1191 (4.3) | |

© 2021 Okubo M et al. *JAMA Network Open.*

| | | | | | | |
|---|---|---|---|---|---|---|
| Other non-public property | 11 (1.0) | 49 (0.6) | | 4 (0.2) | 84 (0.3) | |
| Unknown | 5 (0.5) | 19 (0.2) | | 8 (0.4) | 51 (0.2) | |
| Witnessed collapse | | | 0.259 | | | 0.033 |
| Bystander | 801 (76.2) | 5297 (64.9) | | 784 (35.3) | 9413 (33.8) | |
| None | 225 (21.4) | 2677 (32.8) | | 1383 (62.2) | 17656 (63.4) | |
| Unknown | 25 (2.4) | 184 (2.3) | | 56 (2.5) | 772 (2.8) | |
| Layperson CPR | | | 0.208 | | | 0.133 |
| Yes | 700 (66.6) | 4641 (56.9) | | 906 (40.8) | 13155 (47.3) | |
| No | 330 (31.4) | 3373 (41.3) | | 1270 (57.1) | 14086 (50.6) | |
| Unknown | 21 (2.0) | 144 (1.8) | | 47 (2.1) | 600 (2.2) | |
| Shock delivery before ALS arrival | | | 0.217 | | | 0.027 |
| Yes | 255 (24.3) | 2342 (28.7) | | 22 (1.0) | 306 (1.1) | |
| No | 796 (75.7) | 5816 (71.3) | | 2201 (99.0) | 27535 (98.9) | |
| EMS response time (interval between 9-1-1 call and first EMS arrival), median (IQR), minutes | 5.2 (4.0-6.6) | 5.5 (4.3-7.0) | 0.148 | 5.6 (4.4-7.0) | 5.4 (4.2-7.0) | 0.079 |
| Shock delivery after ALS arrival | | | | | | |
| Yes | 793 (75.5) | 5654 (69.3) | 0.138 | 128 (5.8) | 4483 (16.1) | 0.336 |
| Interval between ALS arrival and shock delivery, median (IQR), minutes | 3.9 (2.6-5.1) | 4.0 (2.5-5.9) | 0.109 | 10.0 (4.4-17.1) | 13.5 (8.5-19.9) | 0.426 |
| Advanced airway management | | | | | | |
| Yes | 455 (43.3) | 6635 (81.3) | 0.853 | 663 (29.8) | 21857 (78.5) | 1.12 |

© 2021 Okubo M et al. *JAMA Network Open.*

| | | | | | | |
|---|---|---|---|---|---|---|
| Interval between ALS arrival and AAM, median (IQR), minutes | 11.0 (7.0-16.9) | 10.5 (7.0-15.0) | 0.116 | 10.0 (6.5-15.3) | 10.5 (7.1-14.7) | 0.04 |
| Departure from the scene | | | | | | |
| Yes | 1001 (95.2) | 6394 (78.4) | 0.515 | 1016 (45.7) | 13753 (49.4) | 0.074 |
| Interval between ALS arrival and departure from the scene, median (IQR), minutes | 19.7 (15.1-25.7) | 24.9 (19.5-31.8) | 0.586 | 19.9 (14.3-26.2) | 25.5 (19.6-32.7) | 0.563 |

ROSC indicates return of spontaneous circulation; TOR, termination of resuscitation; IQR, interquartile range; PEA, pulseless electrical activity; CPR, cardiopulmonary resuscitation; ALS, advanced life support; EMS, emergency medical services; and AAM, advanced airway management.

© 2021 Okubo M et al. *JAMA Network Open.*

**eTable 4.** Characteristics of adult patients with out-of-hospital cardiac arrest with epinephrine and at risk of receiving epinephrine in time-dependent propensity score matched cohort, excluding those who had ROSC or TOR within 5 minutes after ALS EMS arrival

| | Shockable rhythm | | | Nonshockable rhythm | | |
|---|---|---|---|---|---|---|
| | At-risk of receiving epinephrine (n=8145) | Epinephrine (n=8145) | standardized difference | At-risk of receiving epinephrine (n=27827) | Epinephrine (n=27827) | standardized difference |
| Age, median (IQR), y | 65 (55-76) | 65 (55-76) | 0.014 | 67 (55-80) | 68 (55-80) | 0.021 |
| Sex | | | 0.032 | | | 0.006 |
| Male | 6251 (76.7) | 6360 (78.1) | | 17132 (61.6) | 17060 (61.3) | |
| Unknown | 4 (0.0) | 4 (0.0) | | 11 (0.0) | 12 (0.0) | |
| Race | | | 0.053 | | | 0.002 |
| White | 1883 (23.1) | 2069 (25.4) | | 6852 (24.6) | 6828 (24.5) | |
| Non-white | 6262 (76.9) | 6076 (74.6) | | 20975 (75.4) | 20999 (75.5) | |
| Etiology | | | 0.008 | | | 0.009 |
| Cardiac | 7992 (98.1) | 8001 (98.2) | | 25823 (92.8) | 25759 (92.6) | |
| Non-cardiac | 152 (1.9) | 143 (1.8) | | 2004 (7.2) | 2068 (7.4) | |
| Unknown | 1 (0.0) | 1 (0.0) | | | | |
| Initial rhythm | | | | | | 0.025 |
| PEA | N/A | N/A | | 7905 (28.4) | 8223 (29.6) | |
| Asystole | N/A | N/A | | 19922 (71.6) | 19604 (70.4) | |
| Location | | | 0.061 | | | 0.029 |
| Street/highway | 748 (9.2) | 738 (9.1) | | 806 (2.9) | 790 (2.8) | |
| Public building | 141 (1.7) | 167 (2.1) | | 162 (0.6) | 177 (0.6) | |
| Place of recreation | 301 (3.7) | 339 (4.2) | | 386 (1.4) | 320 (1.1) | |
| Industrial place | 182 (2.2) | 178 (2.2) | | 121 (0.4) | 131 (0.5) | |
| Home | 5403 (66.3) | 5275 (64.8) | | 21036 (75.6) | 21067 (75.7) | |
| Farm/ranch | 8 (0.1) | 6 (0.1) | | 23 (0.1) | 26 (0.1) | |
| Healthcare facility | 135 (1.7) | 129 (1.6) | | 550 (2.0) | 564 (2.0) | |

© 2021 Okubo M et al. *JAMA Network Open.*

| | | | | | | |
|---|---|---|---|---|---|---|
| Residential institution | 305 (3.7) | 299 (3.7) | | 3383 (12.2) | 3429 (12.3) | |
| Other public property | 873 (10.7) | 946 (11.6) | | 1197 (4.3) | 1188 (4.3) | |
| Other non-public property | 28 (0.3) | 49 (0.6) | | 94 (0.3) | 84 (0.3) | |
| Unknown | 21 (0.3) | 19 (0.2) | | 69 (0.2) | 51 (0.2) | |
| Witnessed collapse | | | 0.036 | | | 0.017 |
| Bystander | 5192 (63.7) | 5289 (64.9) | | 9206 (33.1) | 9410 (33.8) | |
| None | 2793 (34.3) | 2672 (32.8) | | 17807 (64.0) | 17645 (63.4) | |
| Unknown | 160 (2.0) | 184 (2.3) | | 814 (2.9) | 772 (2.8) | |
| Layperson CPR | | | 0.047 | | | 0.008 |
| Yes | 4450 (54.6) | 4635 (56.9) | | 13048 (46.9) | 13145 (47.2) | |
| No | 3555 (43.6) | 3367 (41.3) | | 14191 (51.0) | 14082 (50.6) | |
| Unknown | 140 (1.7) | 143 (1.8) | | 588 (2.1) | 600 (2.2) | |
| Shock delivery before ALS arrival | | | 0.036 | | | 0.017 |
| Yes | 2452 (30.1) | 2336 (28.7) | | 336 (1.2) | 304 (1.1) | |
| No | 5693 (69.9) | 5809 (71.3) | | 27491 (98.8) | 27523 (98.9) | |
| EMS response time (interval between 9-1-1 call and first EMS arrival), median (IQR), minutes | 5.6 (4.3-7.1) | 5.5 (4.3-7.0) | 0.041 | 5.4 (4.2-7.0) | 5.4 (4.2-7.0) | 0.014 |
| Shock delivery after ALS arrival | | | | | | |
| Yes | 5714 (70.2) | 5646 (69.3) | 0.018 | 4066 (14.6) | 4481 (16.1) | 0.041 |
| Interval between ALS arrival and shock delivery, median (IQR), minutes | 4.1 (2.7-5.9) | 4.0 (2.5-5.9) | 0.026 | 15.3 (8.6-22.9) | 13.5 (8.5-19.9) | 0.166 |
| Advanced airway management | | | | | | |
| Yes | 6258 (76.8) | 6625 (81.3) | 0.111 | 20775 (74.7) | 21845 (78.5) | 0.091 |

© 2021 Okubo M et al. *JAMA Network Open.*

| | | | | | | |
|---|---|---|---|---|---|---|
| Interval between ALS arrival and AAM, median (IQR), minutes | 11.0 (7.0-16.0) | 10.5 (7.0-15.0) | 0.068 | 11.0 (7.0-16.0) | 10.5 (7.0-14.7) | 0.105 |
| Departure from the scene | | | | | | |
| Yes | 6392 (78.5) | 6385 (78.4) | 0.002 | 14421 (51.8) | 13740 (49.4) | 0.049 |
| Interval between ALS arrival and departure from the scene, median (IQR), minutes | 24.9 (19.1-32.3) | 24.9 (19.5-31.8) | 0.002 | 25.3 (19.0-33.3) | 25.5 (19.6-32.6) | 0.021 |

ROSC indicates return of spontaneous circulation; TOR, termination of resuscitation; IQR, interquartile range; PEA, pulseless electrical activity; CPR, cardiopulmonary resuscitation; ALS, advanced life support; EMS, emergency medical services; and AAM, advanced airway management.

**eTable 5.** Outcomes in time-dependent propensity score matched cohort, excluding those who had ROSC or TOR within 5 minutes after ALS EMS arrival

| Outcomes | No (%) patients with outcome/total patients | | Risk ratio (95% CI) |
|---|---|---|---|
| | At-risk of receiving epinephrine | Epinephrine | |
| Shockable rhythms | | | |
| Survival to hospital discharge | 1572/8145 (19.3%) | 1514/8145 (18.6%) | 1.03 (0.95-1.11) |
| Favorable functional outcome at hospital discharge | 1242/8145 (15.2%) | 1176/8145 (14.4%) | 1.02 (0.94-1.11) |
| Prehospital ROSC | 3479/8145 (42.7%) | 3904/8145 (47.9%) | 1.17 (1.12-1.22) |
| Nonshockable rhythms | | | |
| Survival to hospital discharge | 604/27827 (2.2%) | 603/27827 (2.2%) | 1.09 (0.96-1.23) |
| Favorable functional outcome at hospital discharge | 310/27827 (1.1%) | 258/27827 (0.9%) | 0.91 (0.74-1.12) |
| Prehospital ROSC | 6034/27827 (21.7%) | 7880/27827 (28.3%) | 1.33 (1.29-1.38) |

ROSC indicates return of spontaneous circulation; TOR, termination of resuscitation; ALS, advanced life support; and EMS, emergency medical services.

© 2021 Okubo M et al. *JAMA Network Open.*

**eTable 6.** Characteristics of adult patients with bystander witnessed out-of-hospital cardiac arrest with and without epinephrine

| | Shockable rhythm | | | Nonshockable rhythm | | |
|---|---|---|---|---|---|---|
| | No epinephrine (n=1474) | Epinephrine (n=5344) | standardized difference | No epinephrine (n=1151) | Epinephrine (n=9435) | standardized difference |
| Age, median (IQR), y | 62 (53-72) | 65 (55-76) | 0.188 | 70 (58-82) | 71 (58-82) | 0.007 |
| Sex | | | 0.149 | | | 0.175 |
| Male | 1082 (73.4) | 4259 (79.7) | | 606 (52.6) | 5776 (61.2) | |
| Unknown | 1 (0.1) | 2 (0.0) | | 1 (0.1) | 3 (0.0) | |
| Race | | | 0.034 | | | 0.05 |
| White | 387 (26.3) | 1323 (24.8) | | 252 (21.9) | 2266 (24.0) | |
| Non-white | 1087 (73.7) | 4021 (75.2) | | 899 (78.1) | 7169 (76.0) | |
| Etiology | | | 0.058 | | | 0.226 |
| Cardiac | 1443 (97.9) | 5269 (98.6) | | 1002 (87.1) | 8835 (93.6) | |
| Non-cardiac | 31 (2.1) | 74 (1.4) | | 148 (12.9) | 600 (6.4) | |
| Unknown | 0 (0.0) | 1 (0.0) | | 1 (0.1) | 0 (0.0) | |
| Initial rhythm | | | | | | 0.331 |
| PEA | N/A | N/A | | 719 (62.5) | 4361 (46.2) | |
| Asystole | N/A | N/A | | 432 (37.5) | 5074 (53.8) | |
| Location | | | 0.275 | | | 0.153 |
| Street/highway | 159 (10.8) | 483 (9.0) | | 68 (5.9) | 416 (4.4) | |
| Public building | 54 (3.7) | 122 (2.3) | | 14 (1.2) | 90 (1.0) | |
| Place of recreation | 117 (7.9) | 269 (5.0) | | 22 (1.9) | 143 (1.5) | |
| Industrial place | 45 (3.1) | 121 (2.3) | | 2 (0.2) | 56 (0.6) | |
| Home | 738 (50.1) | 3345 (62.6) | | 789 (68.5) | 6827 (72.4) | |
| Farm/ranch | 0 (0.0) | 7 (0.1) | | 1 (0.1) | 12 (0.1) | |
| Healthcare facility | 30 (2.0) | 99 (1.9) | | 49 (4.3) | 281 (3.0) | |
| Residential institution | 45 (3.1) | 127 (2.4) | | 112 (9.7) | 985 (10.4) | |
| Other public property | 261 (17.7) | 721 (13.5) | | 88 (7.6) | 578 (6.1) | |

© 2021 Okubo M et al. *JAMA Network Open.*

| | | | | | | |
|---|---|---|---|---|---|---|
| Other non-public property | 20 (1.4) | 33 (0.6) | | 2 (0.2) | 31 (0.3) | |
| Unknown | 5 (0.3) | 17 (0.3) | | 4 (0.3) | 16 (0.2) | |
| Layperson CPR | | | 0.224 | | | 0.079 |
| Yes | 1060 (71.9) | 3284 (61.5) | | 567 (49.3) | 4880 (51.7) | |
| No | 394 (26.7) | 1972 (36.9) | | 570 (49.5) | 4376 (46.4) | |
| Unknown | 20 (1.4) | 88 (1.6) | | 14 (1.2) | 179 (1.9) | |
| Shock delivery before ALS arrival | | | 0.215 | | | 0.074 |
| Yes | 584 (39.6) | 1614 (30.2) | | 15 (1.3) | 129 (1.4) | |
| No | 890 (60.4) | 3730 (69.8) | | 1136 (98.7) | 9306 (98.6) | |
| EMS response time (interval between 9-1-1 call and first EMS arrival), median (IQR), minutes | 5.1 (4.0-6.6) | 5.6 (4.4-7.1) | 0.192 | 5.5 (4.2-7.0) | 5.5 (4.4-7.2) | 0.056 |
| Shock delivery after ALS arrival | | | | | | |
| Yes | 889 (60.3) | 3686 (69.0) | 0.182 | 80 (7.0) | 1898 (20.1) | 0.392 |
| Interval between ALS arrival and shock delivery, median (IQR), minutes | 3.1 (1.9-4.5) | 3.9 (2.5-5.7) | 0.306 | 9.4 (4.4-18.1) | 13.3 (8.3-19.7) | 0.248 |
| Advanced airway management | | | | | | |
| Yes | 662 (44.9) | 4315 (80.7) | 0.798 | 491 (42.7) | 7655 (81.1) | 0.863 |
| Interval between ALS arrival and AAM, median (IQR), minutes | 10.9 (6.6-16.0) | 10.3 (6.9-15.0) | 0.088 | 10.0 (6.3-15.5) | 10.4 (7.0-14.8) | 0.054 |
| Departure from the scene | | | | | | |
| Yes | 1434 (97.3) | 4354 (81.5) | 0.531 | 808 (70.2) | 5857 (62.1) | 0.172 |

© 2021 Okubo M et al. *JAMA Network Open.*

| | | | | | | |
|---|---|---|---|---|---|---|
| Interval between ALS arrival and departure from the scene, median (IQR), minutes | 19.2 (14.5-25.2) | 24.9 (19.5-31.6) | 0.636 | 20.8 (15.5) | 25.5 (19.7-32.8) | 0.520 |

IQR indicates interquartile range; PEA, pulseless electrical activity; CPR, cardiopulmonary resuscitation; ALS, advanced life support; EMS, emergency medical services; and AAM, advanced airway management

© 2021 Okubo M et al. *JAMA Network Open.*

**eTable 7.** Characteristics of adult patients with bystander witnessed out-of-hospital cardiac arrest out-of-hospital cardiac arrest with epinephrine and at risk of receiving epinephrine in time-dependent propensity score matched cohort

| | Shockable rhythm | | | Nonshockable rhythm | | |
|---|---|---|---|---|---|---|
| | At-risk of receiving epinephrine (n=5326) | Epinephrine (n=5326) | standardized difference | At-risk of receiving epinephrine (n=9418) | Epinephrine (n=9418) | standardized difference |
| Age, median (IQR), y | 65 (55-76) | 65 (55-76) | 0.027 | 70 (58-82) | 71 (58-82) | 0.024 |
| Sex | | | 0.032 | | | 0.025 |
| Male | 4177 (78.4) | 4246 (79.7) | | 5673 (60.2) | 5766 (61.2) | |
| Unknown | 2 (0.0) | 2 (0.0) | | 1 (0.0) | 3 (0.0) | |
| Race | | | 0.038 | | | 0.008 |
| White | 1232 (23.1) | 1318 (24.7) | | 2232 (23.7) | 2263 (24.0) | |
| Non-white | 4094 (76.9) | 4008 (75.3) | | 7186 (76.3) | 7155 (76.0) | |
| Etiology | | | 0.019 | | | 0.019 |
| Cardiac | 5240 (98.4) | 5251 (98.6) | | 8864 (94.1) | 8821 (93.7) | |
| Non-cardiac | 84 (1.6) | 74 (1.4) | | 554 (5.9) | 597 (6.3) | |
| Unknown | 2 (0.0) | 1 (0.0) | | 0 (0) | 0 (0) | |
| Initial rhythm | | | | | | 0.047 |
| PEA | N/A | N/A | | 4134 (43.9) | 4355 (46.2) | |
| Asystole | N/A | N/A | | 5284 (56.1) | 5063 (53.8) | |
| Location | | | 0.078 | | | 0.027 |
| Street/highway | 477 (9.0) | 483 (9.1) | | 412 (4.4) | 415 (4.4) | |
| Public building | 102 (1.9) | 120 (2.3) | | 98 (1.0) | 89 (0.9) | |
| Place of recreation | 238 (4.5) | 269 (5.1) | | 155 (1.6) | 141 (1.5) | |
| Industrial place | 101 (1.9) | 120 (2.3) | | 54 (0.6) | 56 (0.6) | |
| Home | 3427 (64.3) | 3334 (62.6) | | 6826 (72.5) | 6821 (72.4) | |
| Farm/ranch | 13 (0.2) | 6 (0.1) | | 12 (0.1) | 12 (0.1) | |
| Healthcare facility | 109 (2.0) | 97 (1.8) | | 264 (2.8) | 281 (3.0) | |

© 2021 Okubo M et al. *JAMA Network Open.*

| | | | | | | |
|---|---|---|---|---|---|---|
| Residential institution | 151 (2.8) | 127 (2.4) | | 1009 (10.7) | 983 (10.4) | |
| Other public property | 657 (12.3) | 720 (13.5) | | 546 (5.8) | 575 (6.1) | |
| Other non-public property | 27 (0.5) | 33 (0.6) | | 31 (0.3) | 30 (0.3) | |
| Unknown | 24 (0.5) | 17 (0.3) | | 11 (0.1) | 15 (0.2) | |
| Layperson CPR | | | 0.071 | | | 0.027 |
| Yes | 3093 (58.1) | 3277 (61.5) | | 4744 (50.4) | 4870 (51.7) | |
| No | 2139 (40.2) | 1962 (36.8) | | 4484 (47.6) | 4370 (46.4) | |
| Unknown | 94 (1.8) | 87 (1.6) | | 190 (2.0) | 178 (1.9) | |
| Shock delivery before ALS arrival | | | 0.078 | | | 0.024 |
| Yes | 1420 (26.7) | 1606 (30.2) | | 106 (1.1) | 129 (1.4) | |
| No | 3906 (73.3) | 3720 (69.8) | | 9312 (98.9) | 9289 (98.6) | |
| EMS response time (interval between 9-1-1 call and first EMS arrival), median (IQR), minutes | 5.7 (4.4-7.0) | 5.6 (4.4-7.1) | 0.010 | 5.8 (4.4-7.2) | 5.7 (4.4-7.2) | 0.007 |
| Shock delivery after ALS arrival | | | | | | |
| Yes | 3925 (73.7) | 3674 (69.0) | 0.104 | 1694 (18.0) | 1889 (20.1) | 0.053 |
| Interval between ALS arrival and shock delivery, median (IQR), minutes | 4.0 (2.6-5.9) | 3.9 (2.4-5.7) | 0.044 | 15.0 (8.4-22.4) | 13.3 (8.3-19.7) | 0.155 |
| Advanced airway management | | | | | | |
| Yes | 4059 (76.2) | 4302 (80.8) | 0.111 | 7214 (76.6) | 7640 (81.1) | 0.111 |
| Interval between ALS arrival and AAM, median (IQR), minutes | 11.0 (6.7-16.0) | 10.3 (6.9-15.0) | 0.058 | 10.9 (7.0-15.8) | 10.4 (7.0-14.8) | 0.087 |
| Departure from the scene | | | | | | |
| Yes | 4381 (82.3) | 4339 (81.5) | 0.020 | 5870 (62.3) | 5841 (62.0) | 0.006 |

© 2021 Okubo M et al. *JAMA Network Open.*

| | | | | | | |
|---|---|---|---|---|---|---|
| Interval between ALS arrival and departure from the scene, median (IQR), minutes | 25.0 (19.1-32.4) | 24.8 (19.5-31.6) | 0.012 | 25.7 (19.0-33.0) | 25.5 (19.7-32.8) | 0.014 |

IQR indicates interquartile range; PEA, pulseless electrical activity; CPR, cardiopulmonary resuscitation; ALS, advanced life support; EMS, emergency medical services; and AAM, advanced airway management

© 2021 Okubo M et al. *JAMA Network Open.*

**eTable 8.** Outcomes in time-dependent propensity score matched cohort of bystander witnessed out-of-hospital cardiac arrest

| Outcomes | No (%) patients with outcome/total patients | | Risk ratio (95% CI) |
|---|---|---|---|
| | At-risk of receiving epinephrine | Epinephrine | |
| Shockable rhythm | | | |
| Survival to hospital discharge | 1287/5326 (24.2%) | 1178/5326 (22.1%) | 0.97 (0.89-1.05) |
| Favorable functional outcome at hospital discharge | 1059/5326 (19.9%) | 935/5326 (17.6%) | 0.95 (0.87-1.05) |
| Prehospital ROSC | 2531/5326 (47.5%) | 2761/5326 (51.8%) | 1.15 (1.10-1.21) |
| Nonshockable rhythm | | | |
| Survival to hospital discharge | 380/9418 (4.0%) | 323/9418 (3.4%) | 0.94 (0.8.0-1.11) |
| Favorable functional outcome at hospital discharge | 224/9418 (2.4%) | 148/9418 (1.6%) | 0.72 (0.56-0.94) |
| Prehospital ROSC | 2916/9418 (31.0%) | 3708/9418 (39.4%) | 1.31 (1.25-1.37) |

© 2021 Okubo M et al. *JAMA Network Open.*
